# Reduced Adult Survival Estimated in Areas of Decline of Harbour Seal Populations in Scotland

**DOI:** 10.1002/ece3.72349

**Published:** 2025-10-25

**Authors:** M. Arso Civil, S. Tapp, J. Dickens, I. Langley, H. M. Hiley, M. Terrapon, E. Hague, R. C. Hewitt, L. S. Cordes, I. M. Graham, B. J. Cheney, P. M. Thompson, A. Hall, C. E. Sparling

**Affiliations:** ^1^ Sea Mammal Research Unit Scottish Oceans Institute, University of St Andrews St Andrews UK; ^2^ Scottish Association for Marine Science (SAMS) University of the Highlands and Islands Oban UK; ^3^ UHI Shetland, University of the Highlands and Islands Scalloway, Shetland UK; ^4^ Lighthouse Field Station, School of Biological Sciences University of Aberdeen Cromarty UK; ^5^ Norwegian Institute for Nature Research Trondheim Norway; ^6^ Cairngorms National Park Authority Grantown‐on‐Spey UK

**Keywords:** capture–recapture, fecundity, *Phoca vitulina*, photo‐identification, population dynamics, survival, vital rates

## Abstract

Understanding the demographic drivers behind observed changes in wild populations is key to inferring intrinsic and extrinsic causes behind such changes. In Scotland, harbour seal populations have undergone regional declines since the early 2000s. Here, we apply mark–recapture models to photo‐identification data collected during the breeding season at haulout sites representative of three areas with contrasting population trajectories to estimate sex‐specific apparent adult survival and fecundity rates. Apparent adult survival rates were lower at the declining site of Burray, located within the North Coast and Orkney Seal Monitoring Unit (SMU), which has declined by 85% since the mid‐1990s: female survival = 0.844 (95% CI 0.803–0.878) and male survival = 0.826 (95% CI 0.751–0.883) (photo‐ID data collected in 2016–2022). At stable or increasing sites, estimated apparent adult survival rates were higher: at Dunvegan, located in the West Coast SMU, a region that has been generally increasing since monitoring started in the mid‐1990s: female survival = 0.878 (95% CI 0.810–0.924) and male survival = 0.842 (95% CI 0.756–0.902) (photo‐ID data collected in 2016–2022); at Loch Fleet, located in the Moray Firth SMU, which has shown no trend since 2003: female survival = 0.941 (95% CI 0.922–0.956) and male survival = 0.919 (95% CI 0.888–0.942) (photo‐ID data collected 2006–2021). Mark–recapture fecundity rates were generally high at all sites (0.809 to 0.883), with the lowest estimated fecundity at the declining site of Burray. The results indicate that the causes of the decline are likely acting on adult survival, while evidence that a decrease in fecundity is driving the observed declines had less support. The estimated vital rates inform current research into potential causes of the declines and can be incorporated into stage‐structured population dynamic models to investigate whether the hypothesised mechanisms for decline are supported by the data.

## Graphical abstract

We applied mark–recapture models to photo‐ID data from harbour seals at three sites of contrasting population trajectories in Scotland and estimated apparent adult survival and fecundity rates. The results indicate that the causes of the decline are likely acting on adult survival, while evidence that a decrease in fecundity is driving the observed declines had less support.

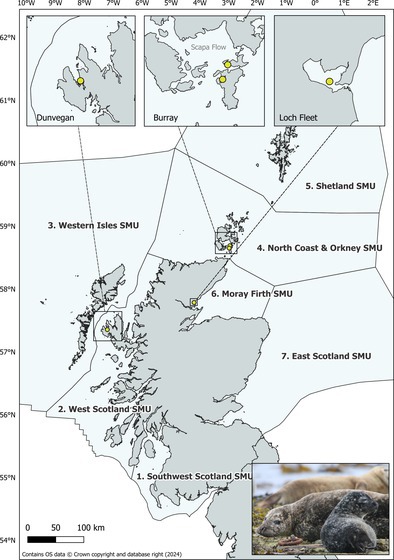

## 1 Introduction

Understanding the demographic drivers behind changes in wildlife abundance is a key question in ecology, conservation biology and wildlife management (Caswell 2001; L. Eberhardt 1985; Williams et al. 2002). Estimation of survival and fecundity is central to understanding demographic fluctuations of animal populations and can help guide efforts to investigate the proximate and ultimate causes of population declines and inform conservation efforts (Caswell et al. 1999; Currey et al. 2011; Kershaw et al. 2021; Pendleton et al. 2006; Schleimer et al. 2019; Warlick et al. 2024). However, inferring causes of population change is complex; declines are rarely driven by a single factor and, even for well‐monitored populations with long‐term data, establishing links between population dynamics and potential intrinsic or extrinsic drivers remains challenging (Bowen et al. 2003; McMahon et al. 2005; Trites 2021).

**FIGURE 1:**
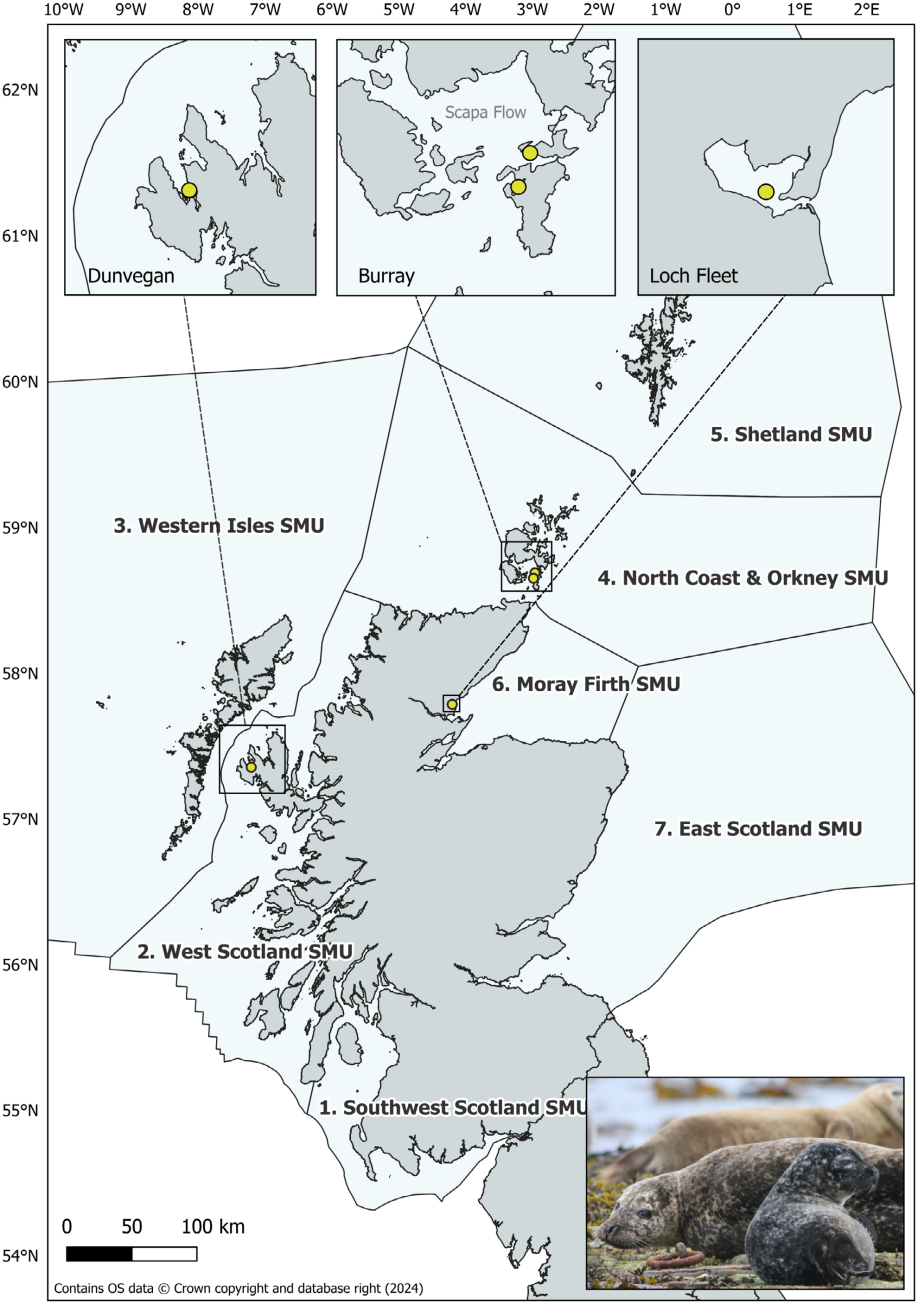
Location of the photo‐ID study sites in Dunvegan, Burray and Loch Fleet (yellow dots) and Seal Monitoring Units (SMUs) in Scotland.

**FIGURE 2:**
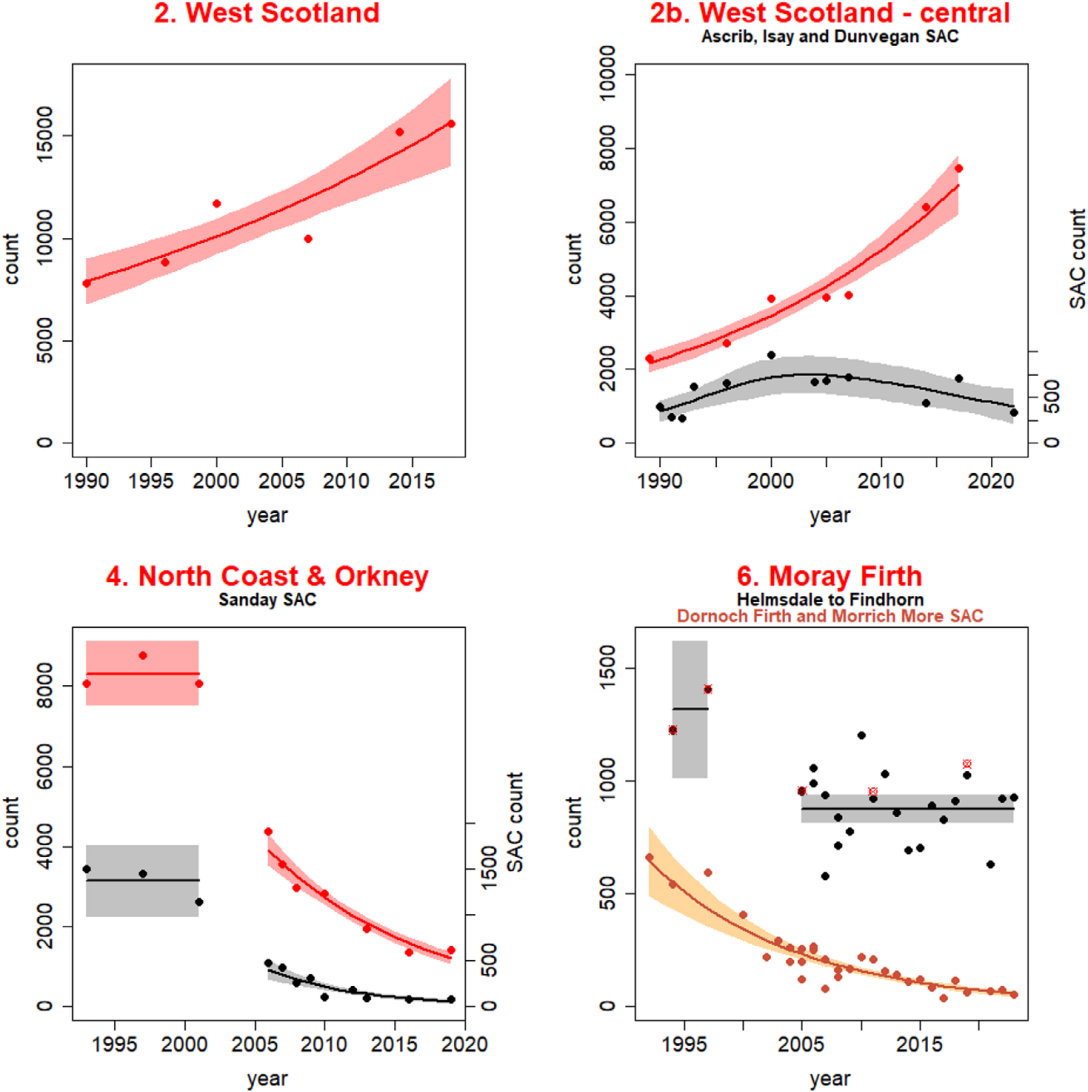
Predicted trend and associated 95% confidence intervals for harbour seal August counts in the three SMUs where photo‐ID effort was conducted (West Scotland SMU, North Coast and Orkney SMU and Moray Firth SMU), and encompassed SACs. Trends are also shown for the central part of West Scotland SMU and encompassed SACs, where the Dunvegan photo‐ID site is located. Note the different axes for the SACs in some plots. Filled circles represent the data points used to fit the trends in Russell et al. (2024). Figure adapted from Russell et al. (2024).

Harbour seals ( *Phoca vitulina* ) are distributed throughout the northern hemisphere from temperate to Arctic regions. Regularly monitored populations show differing population trends, from rapid growth to significant declines (see Blanchet et al. 2021 for a review). The drivers underlying these contrasting trends in abundance remain uncertain, largely due to limited information on variation in survival and fecundity rates. Nevertheless, increases in the proportion of pups in survey counts from populations in the Southern North Sea for example, suggest that recent declines in this region are not a result of reproductive failure (Galatius et al. 2023; SCOS 2024). UK harbour seal populations, which represent around 30% of European harbour seals, have also shown differences in population trends (Thompson et al. 2019; SCOS 2024). The UK populations are subdivided into seal monitoring units (SMU, Figure 1) for the purposes of monitoring and reporting to inform management, although these do not necessarily represent ecological units. Population trends in harbour seals are assessed from count data collected during annual moult aerial surveys in August when a high and relatively stable proportion of the population are hauled out (Lonergan et al. 2013). In Scotland there have been significant changes in regional population trajectories over the last two decades with some areas showing dramatic declines (Thompson et al. 2019). For example, the North Coast and Orkney SMU has declined by 85% since the mid‐1990s; the East Coast SMU has declined by 70% since the late 1990s and the Firth of Tay and Eden Estuary Special Area of Conservation (SAC), which is encompassed within this SMU, has seen a > 90% decline (Russell et al. 2024; Thompson et al. 2019). In contrast, other regions like the West Coast SMU have generally shown stable to increasing trends (depending on the subunit) over the same time period (Thompson et al. 2019) (Figures 1 and 2). A wide range of potential causes for the observed declines in Scotland have been proposed (Hall et al. 2012). Several factors have been ruled out as primary causes, including loss of habitat (SCOS 2020), chemical pollution (Hall and Thomas 2007), epidemics (Lonergan et al. 2010) or bycatch in fishing gear. Other factors remain as likely causes, including predation by grey seals (Brownlow et al. 2016; Langley et al. 2025) and by killer whales (Sutherland 2024), exposure to biotoxins (Hall and Frame 2010; Hall et al. 2024; Jensen et al. 2015), prey availability (Hall et al. 2019; Wilson and Hammond 2019) and competition for prey with other species such as grey seals and harbour porpoise (Langley 2024). These likely causes may not be the same for all areas and multiple causal factors may be acting simultaneously. Nevertheless, the observed declines must have occurred through changes in vital rates or through permanent emigration, although the latter is not considered a major contributing factor in Scotland (Carter et al. 2022; Carroll et al. 2020; McConnell et al. 2017; Olsen et al. 2017; Russell et al. 2015; SCOS 2020). However, previous studies suggest it is unlikely the declines have been solely driven by reproductive failure (fecundity and/or pup survival) (Hanson et al. 2013; Lonergan et al. 2007) and that reduced juvenile and/or adult survival must also be playing a role.

Estimation of vital rates in phocid species, including harbour seals, can be derived from the analysis of dead animals (Bigg 1969; Härkönen and Heide‐Jørgensen 1990), from modelled count data (Reijnders et al. 1997) or mark–recapture models applied to flipper‐tagged, radio‐tagged or branded individuals (Hanson et al. 2013; Härkönen and Harding 2001; Manugian et al. 2017). Because harbour seals can be individually identified from their pelage markings, mark–recapture models can also be applied to photo‐identification (photo‐ID) data for colonies that are accessible (e.g., Cordes and Thompson 2014; Cunningham 2009; Hastings et al. 2012). At the time of this study, life history information for harbour seals in Scotland was only available from photo‐ID studies carried out at two sites in the Moray Firth, NE Scotland, namely Loch Fleet National Nature Reserve (Cordes and Thompson 2014) and the Cromarty Firth (Mackey et al. 2008). Both are included in the Moray Firth SMU, where numbers of harbour seals have been fluctuating with no clear trend since 2003 following a period of decline of > 30% compared to counts in the mid‐1990s (Russell et al. 2024; Thompson et al. 2019). In Scotland, estimates of fecundity are available from a study of progesterone levels in blood and blubber samples from live captured harbour seals in the Moray Firth, West Scotland and North Coast and Orkney SMUs between 2015 and 2018 (Hall et al. 2020).

To help understand the potential drivers behind the observed regional declines in Scotland, long‐term photo‐ID studies were initiated in 2016 at sites within two SMUs with differing trajectories at the time of data collection (the declining North Coast and Orkney SMU, and the stable/increasing West Scotland SMU, see Figure 2 above). Here, we use capture histories derived from these two photo‐ID study sites as well as from the long‐term study in Loch Fleet National Nature Reserve (Moray Firth SMU) to estimate fecundity and apparent survival rates in adult harbour seals to address a recognised data gap in understanding changes in population abundance in different areas. Survival estimation focused on adults (≥ 4 years old). Estimation of juvenile survival from photo‐ID data was not attempted given uncertainty in accurately ageing individuals from photographs (see below), the difficulty of recognising yearlings (1 year old) during the breeding season given their pre‐moult pelage (Thompson and Rothery 1987) and because young harbour seals may display long‐distance movements and natal dispersal from breeding sites (Thompson, Kovacs, and McCocConnell 1994). Estimation of first year pup survival has been carried out in other populations using tagging or branding of pups methods (Bowen et al. 2003; Harding et al. 2005), which were simply not feasible in this study. Additionally, a previous comparative study suggested increased pup mortality was unlikely to be a primary cause driving the Orkney population decline (Hanson et al. 2013) and thus estimation of pup survival was not prioritised. Thus, we first fitted mark–recapture models to the capture histories of seals from the photo‐ID data to estimate overall adult and sex‐specific survival rates. We then used mark–recapture models accounting for reproductive state uncertainty under a robust design framework to estimate fecundity rates and other associated parameters for all adult females and for females known to have given birth to a pup at least once.

## 2 Materials and Methods

### 2.1 Photo‐Identification Data

Harbour seal haulout sites located in regions of Scotland with differing population trends were visited in the spring and summer of 2015 to collect information of their suitability for long‐term photo‐identification studies due to start in 2016. A total of 112 trial sites were visited, located in all SMUs except the Western Isles SMU due to comparatively challenging accessibility (see Arso Civil et al. 2016 for details). Final site selection was informed by historical August aerial survey count data, results from exploratory photo‐ID and ground count surveys in 2015, existing telemetry data, information on usage during both the breeding and moulting seasons, and primarily their suitability to conduct photo‐ID studies.

To represent an area of decline, three nearby haulout sites were selected in Orkney (located within the North Coast and Orkney SMU), one in Widewall Bay and two in the Southwest coast of Burray (Figure 1). The sites (hereafter referred collectively to as Burray) were chosen over others in this SMU for their easy access, suitability for conducting photo‐ID data collection (as shown by earlier observation studies by Rubertus (1983) in southwest Burray), live captures and scat sampling (of interest to other studies occurring alongside the photo‐ID) and having relatively high number of animals during the breeding season (highest compared to any other visited sites in this SMU in 2015). Some of the visited sites in Shetland SMU (another area of population decline) had similar characteristics. However, it was decided to select a site in Orkney because the declining trend for this region was more apparent (85% decline since mid‐1990s and still in decline at an annual rate of 7%–10% during the photo‐ID study period; Hall et al. 2024; Russell et al. 2024) than for Shetland (depleted by 40% compared to counts in 1992 but stable since ~2006; Russell et al. 2024). Widewall Bay (58°48′34″ N, 002°59′05″ W), on the western side of South Ronaldsay, Orkney, is a sheltered enclave where small groups (~10–15 individuals) of harbour seals haul out primarily at a stretch of rocks covered in seaweed and exposed at low tide approximately 100 m away from an observation point on the beach. Seals also haul out in the nearby rocky shores of the bay depending on tide and wind exposure. In Southwest Burray there is a small haulout site on the rocky shore below a hill/cliff area at the end of the village (Langa Taing, 58°50′43″ N, 002°56′48″ W; up to 25 seals may haul out in a day) which harbour seals use mostly at the start of the breeding season (early June). A bigger haulout site is located in Wha Taing (58°50′55″ N, 002°57′49″ W), < 1 km west of Langa Taing, where up to 50 adults and juvenile harbour seals can haul out in a day during the breeding season. The site is located along the sound and consists of a shallow coast and rocky shore covered with seaweed with scattered flat rocks and a terraced rock formation. Photographs can be taken from the coastal path which sits ~50 m away from the seals.

Sites in West Scotland SMU (an SMU representing almost half of the UK harbour seal count and increasing as a whole, although some subunits show no evident trend on the long‐term; Russell et al. 2024) and in Southwest Scotland SMU (increasing and at the highest levels of the time series of August count aerial surveys; Russell et al. 2024) were generally difficult to access from land, with many sites located in skerries too far from land to obtain individual photo‐ID data using digi‐scoping. In southeastern Loch Dunvegan (57°27′21″ N, 006°36′13″ W), located within the Ascrib, Isay and Dunvegan SAC in the central subunit of West Scotland SMU, harbour seals haul out at a complex of skerries, islets and undisturbed mainland rocky shores in large numbers (up to 190 adults and juveniles haul out in a day during the breeding season, although numbers have varied through the study period, see Table 1 and discussion). The site (hereafter referred to as Dunvegan, Figure 1) was chosen because it can be regularly accessed by 3‐m tourist clinker boats that have been operating in this area since the 1970s. Seals show minimal disturbance behaviour suggesting they are somewhat acclimatised to the boats, and a previous photo‐ID study was conducted here in 2005 (Cunningham 2009).

Photo‐ID data were collected at Burray and Dunvegan between 2016 and 2022 (with no data collection in 2020 due to COVID 19). At Burray, this period overlapped with a time of population decline in the North Coast and Orkney SMU. At Dunvegan, it overlapped with a time of population increase in the wider West Scotland SMU and central subunit, with no significant trend in the Ascrib, Isay and Dunvegan SAC (Russell et al. 2024) where Loch Dunvegan is located. Additionally, photo‐ID data have been collected at Loch Fleet National Nature Reserve (hereafter referred to as Loch Fleet) as part of a separate long‐term study since 2006 (Cordes and Thompson 2014, 2015), from which 2006 to 2021 data were used for this study (Figure 1). This overlaps with a period of no clear trend in the wider Moray Firth SMU since 2003 after a period of decline (Thompson et al. 2019). Loch Fleet is a large tidal basin surrounded by dunes, saltmarsh and pine woods. At low tide, a series of intertidal sand banks are exposed, where harbour seals haul out in large numbers (up to 170 adult and juvenile seals haul out in a day during the breeding season, Table 1). Photographs can be taken from an observation point on land, ≥ 130 m away.

Photo‐identification data collection and processing protocols for Burray and Dunvegan were adapted from those used at the already established Loch Fleet study. For all sites, photo‐identification data were collected in June and July, during the breeding season in Scotland when harbour seals have their pups. Sites were visited around low tide when seals are more likely to haul out, with photographs taken using a DSLR camera attached to a scope (Swarovski ATS 80 with × 20–60 eyepiece and TLS‐APO 30 mm) at Burray and Loch Fleet from observation points on land. At Dunvegan a DSLR camera with a 80–400 mm zoom lens was used from the clinker boats. At Burray, all haulout sites were visited at least once every 3 days at the start of the season in early June and daily or every 2 days following the sighting of the first pup. Surveys started approximately 1 h before low tide and lasted 2–4 h. Changes in tidal exposure forced redistribution of animals and provided additional opportunities for photographs as animals moved (see Arso Civil et al. 2021 for details) (Table 1). In Loch Fleet, surveys started at low tide and lasted 3–5 h. At Dunvegan, a researcher joined the boat trips 2–4 times per week, weather‐ and boat‐availability‐dependent, with trips lasting between 30 min and 1 h. The survey route depended on weather conditions and presence of other boats, but equal coverage of the area was attempted and seals were photographed systematically from one side of each skerry to the other as the boat was travelling around, to reduce the possibility of heterogeneity in capture probability. In all areas, surveys were planned to occur in dry weather conditions as much as possible. When using the digiscope system, relatively calm days were preferred to avoid equipment shake with the wind which may result in blurry photographs. Hazy conditions in hot and calm days were avoided if possible (or photo‐ID effort aborted) as it compromised the ability of obtaining good quality photos due to heat distortion, although count data and observations could still be collected. The number of seals present at the haulout site(s) were counted during each photo‐ID survey, generally at the start, half‐way and end of each survey.

For all sites, photographs were taken of the right and left sides of the head and neck region primarily, to build a catalogue of identified seals based on their distinct pelage pattern. At Dunvegan, seals often looked towards the boat (and photographer) and thus front‐side photographs were most frequently obtained. To minimise potential bias from photographic quality (e.g., Friday et al. 2008) due to different photo‐ID set up, the same quality grading protocol (adapted from Cunningham 2009) was applied to all data. Photographs were graded from poor (1) to excellent (4) photographic quality based on the angle, clarity of pattern and focus of the head and neck area. Only identifications from photographs graded as 3 or 4 (good to excellent) were used in any subsequent analysis. Photographs from Loch Fleet were identified and matched manually as part of the long‐term project by the team of researchers at the University of Aberdeen. In Burray and Dunvegan, a freely available computer‐assisted pattern matching software (Wild‐ID; Bolger et al. 2012; Langley et al. 2021) was used to help manual matching, by narrowing down the initial potential matches to each photograph (Langley et al. 2021). Photographs of the ventral aspect of seals were used to determine sex, with females also identified if observed nursing a pup. Field observations and photographs were used to assign pups to mothers on each survey day, based on close association with a pup and observed suckling. In Burray and Dunvegan, efforts were made to identify pregnancies from photographs and field observations when possible, to have a record of which females were seen pregnant each season.

The estimation of survival and fecundity rates presented here focused on adult seals, defined as individuals ≥ 4 years old. In Loch Fleet, seals were classed as adults once they had been seen for at least 4 years and, for females, also since the first known pup (whichever occurred first). In Burray and Dunvegan, where only 6 years of data were collected and discarding 4 years of data was not an option, identified seals were classed into four broad age classes based on their body size and pelage characteristics: pups (small size, black and white new pelage, associated to/nursing from an adult female); yearlings (1 year old, similar small size to a weaned pup but with characteristic pre‐moult pale un‐patterned pelage; Thompson and Rothery 1987); juveniles (2–3 years of age, larger than yearlings but smaller than fully developed adults, with generally less defined pattern markings compared to adults); or adults (≥ 4 years old). While it is not possible to accurately determine the age of harbour seals from photographs (Thompson and Rothery 1987), the broad age classes were informed by photographs of seals of known age from the three study areas either because they were photo‐identified since birth or were aged from growth layer groups in teeth during capture–release studies (Hall et al. 2019). While different researchers were involved in the collection of photo‐ID data during the study, the same experienced researcher was always involved in classifying individuals into age‐groups for all study years. Potential biases from ageing seals from photographs on estimated vital rates are addressed in the discussion.

### 2.2 Sighting Histories and Goodness‐of‐Fit Tests

Prior to model fitting, sighting and reproductive histories were prepared for each of the study sites. For Burray and Loch Fleet, all identified adult seals were included in the analysis of survival and fecundity as they all had matched right (R) to left (L) side photographs of the head/neck. At Dunvegan, a proportion of catalogued seals had no R‐ or L‐side photograph, but most were known from front (F) side photographs. To minimise the inclusion of false negatives in the data (i.e., the same seal is given two or more different IDs), sighting histories were generated exclusively from F‐side photographs for survival estimation in Dunvegan (e.g., Mackey et al. 2008). For fecundity estimation in Dunvegan, all sides were used, as exploration of the catalogue showed that most identified adult females (226 out of 232, 97%) were photographed at least from the F‐side, minimising the chances of false negatives.

Package R2ucare (Gimenez et al. 2018) in R (R Core Team 2023) was used to run goodness‐of‐fit (GOF) tests to assess the fit of general Cormack‐Jolly‐Seber (CJS) models (Cormack 1964; Jolly 1965; Seber 1965) to each dataset and to check on the basic assumptions regarding the probability of capture (Lebreton et al. 1992). Specifically, R2ucare was used to identify unequal survival or recapture probabilities due to transients (test 3SR; Pradel et al. 1997) or trap‐dependence (trap‐happiness or trap‐shyness, test 2CT; Pradel 1993). Transients were identified as individuals seen only once, and thus having a survival probability of zero after their first capture, and were accounted for by fitting time‐since‐marking (TSM) models in which survival estimates are generated for the “transient” animals (i.e., survival of individuals in the year following the first capture) and for “non‐transient” animals (i.e., survival in all subsequent years; parameter of interest). Trap‐dependence may emerge for example if individuals have higher recapture probabilities because they spend more time in more accessible parts of a study area (Pradel and Sanz‐Aguilar 2012), which in this case might just be a preference for a more or less accessible location for photo‐ID within the haulout site. Trap‐dependence was accounted for when necessary by incorporating an individual time‐varying covariate to allow capture probabilities to vary depending on whether an individual had been captured in the previous occasion (i.e., year) or not (Huggins 1989). Finally, the over‐dispersion factor (*ĉ*) was estimated and used to adjust model statistics and confidence intervals around estimated parameters when *ĉ* > 1.

### 2.3 Survival Estimation

Sighting histories of adults (≥ 4 years old) were constructed as strings of 1 and 0 s to describe if each seal was seen (1) or not seen (0) each year, between 2016 and 2022 in Burray and Dunvegan with a 2‐year interval between 2019 and 2021 due to no data collection in 2020; and between 2010 and 2021 in Loch Fleet, excluding the first 4 years of data which only contained females given seals only enter the sighting history in Loch Fleet when they have been seen for +4 years, or for females the year first seen with a pup, whichever occurred first. Apparent survival rates (*φ*) and recapture probabilities (*p*) were estimated using mark–recapture open population CJS models. Sighting histories were also prepared for a subset of the data including only sexed animals to estimate sex‐specific survival rates. Sex remained undetermined for a number of catalogued seals, especially in Dunvegan. To avoid positive bias in survival rates of known‐sexed seals, the ad hoc approach described in Nichols et al. (2004) was implemented, in which individuals only enter the sighting history the year they are sexed, and all previous sightings are discarded. Apparent survival rate is the product of (a) the probability an individual survives from occasion *t* to *t* + 1 (true survival) and (b) the probability that the individual is present in the study area (site fidelity). Apparent survival probabilities were modelled as constant (.), sex‐specific (*sex*) and with annual variability (*t*) or with a linear trend (*T*), as an additive or interaction effect with sex. Recapture probabilities were allowed to be constant (.), to vary between years (*t*) and/or by sex (*sex*). Following Gimenez et al. (2018), models were fitted with added trap‐dependence and/or transience depending on the outcome of the GOF tests.

Model selection was based on the Akaike Information Criterion adjusted for small sample size (AICc) (Anderson et al. 1994) and on the Quasi Akaike Information Criterion (QAICc) (Burnham and Anderson 2002) in the presence of over‐dispersion. When several models were well supported, model averaging was used. Models were constructed in R (version 4.2.2) with package RMark (Laake 2013), which runs models in program MARK (White and Burnham 1999).

### 2.4 Fecundity Estimation

Fecundity rates were estimated based on the sighting and reproductive histories of adult (≥ 4 years old) females from Burray and Dunvegan (period 2016 to 2022) and Loch Fleet (period 2006 to 2021).

Models were also run for a subset of the data including females only from the first known pup (i.e., sightings in years before the first known pup were set to zero in the capture histories), hereafter referred as “known reproductive females”. This was done to obtain a fecundity rate for known reproductive females, given the uncertainty in reproductive state of younger 3‐ to 5‐years old female harbour seals (Boness et al. 1994; Cordes and Thompson 2014; Härkönen and Heide‐Jørgensen 1990).

Fecundity rates were estimated using open robust design multistate models with state uncertainty and seasonal effects (RDMSOpenMCSeas2 in RMark package; data type 184 in MARK). This is a modification of a similar model (RDMSOpenMCSeas, data type 142; White 2014) that has been applied to estimate fecundity in other marine mammal species (e.g., Cheney et al. 2019). Sighting and reproductive histories of adult females were constructed for a 6‐week period from the 8th June to 16th July every summer to encompass the breeding season (when harbour seals in Scotland have their pups). Under the robust design, years (each breeding season) represent primary occasions, and the 6 weeks within each breeding season, are secondary occasions. Females were assigned one of two states in each of the weeks: breeder (B) for females seen with a pup, or unknown (u), for females seen without a pup. This unknown state will comprise a mix of non‐breeders and breeders where the pup is not photographed, obscured by the female, separated from the female or dead. The model uses the sightings of females (with or without a pup) within each season to determine the probability of correctly classifying a female as a breeder (*δ* ^B^) (Cordes and Thompson 2014; Kendall et al. 2003). The model allows females to arrive and depart at different times through the breeding season and their state to change from non‐breeder to breeder and vice versa within the season (seasonal effect). The model estimates a number of parameters of interest to this particular study: the proportion of females with a pup (*ω* ^B^, unconditional reproductive rate hereafter referred as fecundity rate). The transition probability from a non‐breeder to a breeder between years (*Ψ* ^*NB*^) and the probability of staying a breeder between years (*Ψ* ^*BB*^), which are conditional reproductive rates, because they are conditional upon the female's state in the previous year. Other parameters include the probability that the attribute to assign the breeder state (i.e., pup) has appeared (*α*, the pupping probability), and that it is present (c, with a decrease in c indicating weaning of the pup), the probability of apparent survival of breeders and non‐breeders between years (*S*_B_, *S*_N_), the probability of recapture (*p*), the proportion of the females released or entering the capture matrix in a certain state (as a breeder or non‐breeder) during each primary period (year) (*π*), and the probabilities of entering (*e*) or remaining (*φ*) in the study area.

To avoid running all possible parameter combinations, a step‐forward model selection approach was implemented. In each step, a set of models was fitted with one parameter allowed different formulations regarding time and state (see below), while keeping all other parameters fixed. The most supported formulation for such parameter (based on AICc) was selected and applied to the next set of models, until all parameters had been formulated. Parameters were fitted as constant (.), state‐ (*s*) and/or time‐dependent. To avoid over‐parameterisation, linear or quadratic trends across primary (*T* and *T* ^2^) or secondary (*t* and *t* ^2^) occasions were preferred, although weekly or annual variation was allowed. It was assumed that non‐breeders could not be wrongly classified as breeders as each pup was correctly assigned to each female, and therefore δ^N^ was fixed to 0. Survival was modelled constant or with a time trend, with or without variation between breeders and non‐breeders. Fecundity rate (*ω* ^B^) was modelled constant, varying by year or with a linear trend over time. Recapture probabilities (*p*) were modelled either as constant, with a linear or quadratic trend within the breeding season (secondary occasions) and varying or not between states (breeder vs. non‐breeder). Models were also fitted with recapture probability constant for non‐breeders but with a linear or quadratic trend for breeders, to reflect potential differences in the probability of seals being at the haulout site at different times of the breeding season due to breeding status (Boness et al. 1994). For the probabilities of entering and remaining, models were fitted with a linear or quadratic trend and allowed to differ by state. For the pupping probability and probability that the pup is still present (*α* and *c*, respectively) models were fitted with a linear or quadratic trend or allowed to vary within the season. For both survival and fecundity models, when a time trend (*T*) was included, the trend (positive or negative) was considered significant if the 95% confidence interval around the beta (β) estimate of the parameter excluded zero (Cheney et al. 2019).

## 3 Results

### 3.1 Photo‐ID Effort

Photo‐ID effort varied between years but was similar in Burray (32–54 days per season; $\bar{x}$ = 44 ± 8 SD) and Loch Fleet (14–53 days per season; $\bar{x}$ = 41 ± 10 SD), with a smaller number of trips conducted per season in Dunvegan (16–29 days per season; $\bar{x}$ = 23 ± 4 SD) (Table 1). Ground counts of seals during photo‐ID surveys were smallest in Burray with a maximum of 51 seals counted in a haulout site per trip ($\bar{x}$ = 20 ± 11 SD), compared to Dunvegan (2–187 seals counted per trip, $\bar{x}$ = 61 ± 41 SD) and Loch Fleet (82 ± 28 SD). Ground counts declined in both Burray and Dunvegan over the study period but increased in Loch Fleet (*p*‐value < 0.001 for all sites) (Table 1).

**TABLE 1:** Summary of photo‐ID effort in each study site per breeding season (June and July).

Study site	Year	# photo‐ID days	# seals counted at haul out site (x̄ ± SD)	# seals counted at haul out site (range)	# IDs (adults)	# IDs males (adults)	# IDs females (adults)	# IDs (juveniles)	# Proportion of pregnant females not seen later in the season	# Mum‐pup pairs
Burray	2016	53	22 ± 9 SD	0–40	89	21	67	15	5.9% (*n* = 4)	42
	2017	48	23 ± 12 SD	0–50	90	21	65	20	4.6% (*n* = 3)	41
	2018	54	23 ± 10 SD	4–41	84	19	63	15	9.5% (*n* = 6)	33
	2019	44	24 ± 12 SD	0–51	77	15	59	19	1.7% (*n* = 1)	29
	2021	32	15 ± 8 SD	0–34	66	11	48	17	12.5% (*n* = 6)	26
	2022	37	14 ± 8 SD	0–31	62	15	45	5	0% (*n* = 0)	30
Dunvegan	2016	16	74 ± 47 SD	19–161	225	86	107	37	3.8% (*n* = 4)	61
	2017	20	84 ± 44 SD	18–157	303	105	138	75	6.5% (*n* = 9)	81
	2018	25	89 ± 39 SD	39–187	332	118	147	41	3.4% (*n* = 5)	69
	2019	29	64 ± 30 SD	12–149	353	121	160	2	10.0% (*n* = 16)	80
	2021	25	26 ± 20 SD	2–69	213	44	133	0	13.5% (*n* = 18)	53
	2022	24	39 ± 28 SD	9–131	231	74	108	0	37.0% (*n* = 40)	48
Loch fleet	2006	45	45 ± 12 SD	9–64	33	0	33	—	—	32
	2007	48	45 ± 11 SD	9–66	38	0	38	—	—	32
	2008	48	57 ± 12 SD	32–79	51	0	51	—	—	48
	2009	46	63 ± 13 SD	32–85	59	0	59	—	—	43
	2010	47	74 ± 15 SD	41–111	96	26	70	—	—	49
	2011	48	78 ± 17 SD	35–110	92	27	65	—	—	40
	2012	52	86 ± 18 SD	44–124	103	31	72	—	—	45
	2013	53	88 ± 20 SD	32–129	113	41	72	—	—	44
	2014	35	85 ± 18 SD	46–131	117	39	78	—	—	54
	2015	43	98 ± 21 SD	60–143	118	35	83	—	—	55
	2016	43	96 ± 20 SD	47–138	139	54	85	—	—	58
	2017	47	111 ± 26 SD	59–156	145	61	84	—	—	58

	2018	44	103 ± 23 SD	62–150	155	65	90	—	—	68
	2019	33	97 ± 21 SD	57–138	153	66	87	—	—	61
	2020	14	107 ± 18 SD	70–145	158	69	89	—	—	68
2021	19	116 ± 30 SD	57–171	172	71	101	—	—	73

*Note:* Number of photo‐ID effort days and average number of seals (non‐pup) counted during photo‐ID surveys, with associated standard deviation (SD) and range (min–max). Photo‐identification outputs are shown based on quality 3 and 4 photographs as the number of total IDs (adults), the number of identified adult males and females, the number of seals classified as juveniles in Burray and Dunvegan, the number of pregnant females not seen later in the season and the number of mum‐pup pairs. Adult is defined here as ≥ 4 years old to construct capture histories for the estimation of survival and fecundity rates. Note that for Loch Fleet, the years 2006–2010 only contain adult females given how seals enter the analysis (i.e., seen for at least 4 years or since the first known pup for females). Data on # juveniles and # pregnant females not seen later in the season (i.e., might have left the study area) were not available from the long‐term study in Loch Fleet.

The number of uniquely photo‐identified adults also varied by season and site (Table 1). Burray resulted in the smallest catalogue (62–90 adult IDs per season) with decreasing number of identified adults (*p*‐value < 0.001), juveniles (*p*‐value = 0.23) and of mum‐pup pairs (*p*‐value < 0.05), over the study period. Dunvegan had a larger catalogue although the number of photo‐identified adults identified every season decreased from an average of 303 (±56 SD) in 2016–2019 to 222 (±13 SD) in 2021–2022 (*p*‐value = 0.55). The number of individuals classed as juveniles in Dunvegan also decreased (*p*‐value = 0.06), from 51 (±21 SD) juveniles identified on average every season in 2016–2018, to only 2 individuals identified in 2019 and none in 2021 or 2022. In Loch Fleet the number of adult IDs per year increased from 2006 to 2021, as did the number of mum‐pup pairs (*p*‐value < 0.001) (Table 1). At Burray and Dunvegan, identified pregnancies were followed through photo‐ID efforts when possible. A proportion of identified pregnant females seen at the start of each season were then not seen later on, suggesting they had left the study area before pupping (Table 1). In Burray this proportion ranged from 1.7% to 12.5% and declined over time (*p*‐value = 0.84). In Dunvegan it ranged from 3.4% to 37%, significantly increasing through the study period (*p*‐value < 0.05).

### 3.2 Survival Rates

At Burray, a total of 156 adult seals (≥ 4 years old) were identified between 2016 and 2022, including 102 females, 41 males and 13 individuals of unknown sex. GOF tests indicated an overall good fit (*χ* ^2^ = 18.93, df = 12, *p* = 0.09) with a small amount of over‐dispersion (*ĉ* = 1.578), used to correct model selection. Two top models received support, both with constant recapture probability (*p*_ad_ = 0.887, 95% CI 0.824–0.929), and where apparent survival was modelled as either constant (top model; S_ad_= 0.830 95% CI: 0.782–0.869) or with a linear trend (non‐significant as the 95% CI around the *β*‐estimate overlapped with zero). The dataset of sexed individuals (102 females and 41 males) showed under‐dispersion (*ĉ* = 0.624) and a good fit (*χ* ^2^ = 13.722, df = 22, *p* = 0.91). The top two models (ΔAICc < 1) included constant or sex‐specific survival, indicating some support for survival being different between females and males. Model‐averaged estimates of apparent survival were 0.844 (95% CI: 0.803–0.878) for females (*S*_F_) and 0.826 (95% CI: 0.751–0.883) for males (*S*_M_). Recapture probabilities were 0.938 (95% CI: 0.890–0.966) for females (*p*_F_) and 0.711 (95% CI: 0.567–0.822) for males (*p*_M_) (Table 2).

**TABLE 2:** Estimates of parameters of interest from the survival and fecundity analyses at three study sites.

	Burray, North Coast and Orkney SMU			Dunvegan, West Scotland SMU			Loch Fleet, Moray Firth SMU		
	Estimate	SE	95% CI	Estimate	SE	95% CI	Estimate	SE	95% CI
**Survival models**									
*S*_ad_	0.830	0.022	0.782–0.869	0.938	0.027	0.858–0.974	0.932	0.007	0.917–0.945
*S*_F_	0.844*	0.019	0.803–0.878	0.878*	0.029	0.810–0.924	0.941	0.009	0.922–0.956
*S*_M_	0.826*	0.033	0.751–0.883	0.842*	0.037	0.756–0.902	0.919	0.014	0.888–0.942
*p*_ad_	0.887	0.026	0.824–0.929	0.341 (0.256–0.437) to 0.617 (0.457–0.756)			0.797 (0.713–0.861) to 0.884 (0.841–0.916)		
*p*_F_	0.938*	0.018	0.890–0.966	0.562 (0.435–0.681) to 0.882 (0.800–0.933)*			0.797 (0.711–0.863) to 0.883 (0.837–0.917)*		
*p*_M_	0.711*	0.066	0.567–0.822	0.289 (0.184–0.422) to 0.702 (0.549–0.820)*			0.800 (0.707–0.869) to 0.886 (0.834–0.924)*		
**Fecundity** ≥ **4 years old**									
*Ψ* ^*BB*^	0.798	0.044	0.696–0.872	0.910*	0.043	0.784–0.966	0.895	0.030	0.818–0.941
*Ψ* ^*NB*^	0.434	0.064	0.316–0.560	0.215*	0.104	0.076–0.477	0.360	0.082	0.219–0.530
*ω* ^B^	0.631	0.028	0.574–0.685	0.728	0.038	0.646–0.796	0.769	0.021	0.725–0.807
*δ* ^B^	0.895	0.018	0.853–0.926	0.825*	0.034	0.749–0.882	0.988	0.006	0.969–0.995
*p* ^B^	0.715 (0.657–0.767) to 0.993 (0.975–998)			0.741 (0.102–0.541) to 0.509 (0.354–0.663)			0.658 (0.585–0.725) to 0.891 (0.870–0.909)		
*p* ^N^	0.580 (0.485–0.670) to 0.987 (0.954–0.997)			0.471	0.036	0.402–0.541	0.606 (0.513–0.629) to 0.825 (0.760–0.875)		
*S* ^B^	0.843	0.019	0.801–0.877	0.717 (0.607–0.807) to 0.964 (0.928–0.982)			0.951 (0.903–0.976) to 0.955 (0.902–0.980)		
*S* ^N^							0.891 (0.750–0.957) to 0.899 (0.777–0.958)		
**Fecundity known reproductive females**									
*Ψ* ^*BB*^	0.754*	0.052	0.638–0.842	0.714*	0.046	0.616–0.795	0.883	0.015	0.850–0.910
*Ψ* ^*NB*^	0.577*	0.122	0.339–0.784	0.695*	0.117	0.437–0.870	0.607	0.058	0.490–0.713
*ω* ^B^	0.809	0.033	0.737–0.865	0.883	0.025	0.823–0.924	0.872	0.012	0.847–0.894
*δ* ^B^	0.895*	0.022	0.842–0.932	0.830*	0.025	0.775–0.873	0.992	0.003	0.984–0.997
*p* ^B^	0.665 (0.568–0.749) to 0.936 (0.898–0.951)*			0.458 (0.358–0.563) to 0.704 (0.660–0.744)*			0.665 (0.622–0.705) to 0.904 (0.891–0.915)		
*p* ^N^	0.543 (0.398–0.680) to 0.897 (0.834–0.938)*			0.458 (0.358–0.563) to 0.666 (0.407–0.853)*			0.784	0.019	0.744–0.820
*S* ^B^	0.835	0.028	0.772–0.883	0.709 (0.615–0.788) to 0.961 (0.930–0.979)			0.951 (0.917–0.972) to 0.980 (0.956–0.991)		
*S* ^N^							0.819 (0.689–0.903) to 0.918 (0.821–0.965)		

*Note:* Estimates of parameters of interest from the survival and fecundity analyses at three study sites. Survival and probabilities of capture estimates are shown for ≥ 4 years old seals as a single parameter (S_ad_, p_ad_, respectively), and sex‐specific (S_F_, S_M_, and p_F_, p_M_, respectively). Probabilities of recapture (p) are shown as constant or as the range of annual estimates based on the most supported model. Relevant fecundity related parameters from the robust design multistate models with state uncertainty and seasonality are shown for all ≥ 4 year‐old harbour seal females and for known reproductive females separately, where Ψ^BB^ = probability of staying a breeder between years and Ψ^NB^ = transition probability from a non‐breeder to a breeder between years (conditional reproductive rates); ω^B^ = fecundity rate (unconditional reproductive rate); δ^B^ = probability of correctly classifying a female as a breeder; p^B^ = recapture probability for breeders; p^N^ = recapture probability for non‐breeders. Associated standard error (SE) and 95% Confidence Interval (CI) are shown. Averaged parameters over 2 or more well‐supported models are marked with an asterisk (*).

At Dunvegan, 525 seals (≥ 4 years old) were identified between 2016 and 2022, including 216 females, 172 males and 137 seals of unknown sex, and showed a good fit (*χ* ^2^ = 12.878, df = 8, *p* = 0.12) once transience and trap‐dependency were accounted for with a remaining over‐dispersion of *ĉ* = 1.609. Two top models received support (ΔQAICc < 2), one with constant survival and the second one with a linear trend on survival (not significant based on the β‐estimate). The top model estimated adult apparent survival (*S*_ad_) at 0.938 (95% CI: 0.858–0.974) and had time‐varying recapture probabilities (*p*_ad_) ranging from 0.341 (95% CI: 0.256–0.437) to 0.617 (95% CI: 0.457–0.756). GOF tests on the dataset of sexed individuals (216 females and 172 males) indicated the presence of transience in females only, which was incorporated into the models. Once it was accounted for, data showed a good fit (*χ* ^2^ = 26.287, df = 18, *p* = 0.09) with some over‐dispersion (*ĉ* = 1.46), which was used to adjust model selection. Three models received support (ΔQAICc < 3), with survival modelled as constant, varying by sex or by sex with a linear trend over time, and time‐ and sex‐varying recapture probabilities. Model averaging of the top two models (excluding the model with a linear trend fitted to survival) resulted in a model‐averaged apparent survival for females (*S*_F_) of 0.878 (95% CI: 0.810–0.924) and for males (*S*_M_) of 0.842 (95% CI: 0.756–0.902) (Table 2). Recapture probabilities for males (*p*_M_) and females (*p*_F_) from the most supported ad hoc sex‐specific survival models decreased over time but not significantly (*p*‐value > 0.35).

At Loch Fleet, 282 adult seals (≥ 4 years old) were identified between 2010 and 2021, including 157 females, 119 males and 6 seals of unknown sex. GOF tests showed signs of trap‐dependency which once accounted for in models resulted in a good fit (*χ* ^2^ = 24.336, df = 27, *p* = 0.611) and *ĉ* = 0.901. The top two models (ΔAICc < 3) included apparent survival (*S*_ad_) with a linear trend (non‐significant based on β‐parameter) or constant (0.932, 95% CI: 0.917–0.945), with recapture probability (*p*_ad_) ranging from 0.797 (95% CI: 0.713–0.861) to 0.884 (95% CI: 0.841–0.916) between years. The sex‐specific ad hoc survival models had a good fit (*χ* ^2^ = 29.581, df = 46, *p* = 1) and *ĉ* = 0.643, once trap‐dependence was accounted for. Four models were within 2 AICc scores, with survival and recapture probabilities modelled as constant or varying by sex. Model‐averaged estimates of apparent survival were 0.941 (95% CI: 0.922–0.956) for females (*S*_F_) and 0.919 (95% CI: 0.888–0.942) for males (*S*_M_) (Table 2).

### 3.3 Fecundity Rates

At Burray, 79 of the 102 adult females (≥ 4 years old) pupped at least once. GOF tests indicated an overall good fit (*χ* ^2^ = 13.137, df = 11, *p* = 0.28) of the adult female dataset and *ĉ* = 1.1. The most supported model (Table 3) estimated a fecundity rate (*ω* ^B^; proportion of females with a pup each year) of 0.631 (95% CI: 0.574–0.685). The probability of pupping following a year with no pup (*Ψ* ^*NB*^) was 0.434 (95% CI: 0.316–0.560) while the probability of pupping following a year with pup (*Ψ* ^*BB*^) was 0.798 (95% CI: 0.696–0.872). Data from known reproductive females also had a good fit (*χ* ^2^ = 13.377, df = 13, *p* = 0.146) with a small level of over‐dispersion (*ĉ* = 1.486) used to correct model selection. Three models were within 1 ΔQAICc, differing on how fecundity rate (*ω* ^B^) was modelled: constant (0.809, 95% CI: 0.737–0.865), varying by year (ranging 0.722 to 0.99) or with a trend (significant negative trend based on β‐parameter values, with estimates ranging from 0.869 to 0.715). Model‐averaged probabilities of pupping were 0.577 (95% CI: 0.339–0.784) after a year with no pup (*Ψ* ^*NB*^) and 0.754 (95% CI: 0.638–0.842) following a year with pup (*Ψ* ^*BB*^) (Table 2).

**TABLE 3:** Model selection to estimate reproductive rates of ≥ 4‐year old females and from known reproductive females (from first recorded pup).

	npar	ΔAICc/ΔQAICc	Weight	*ĉ*
Burray ≥ 4‐year old females				
*S*(.) *Ψ*(*s*,.) *π* ^*B*^(.) *ω* ^*B*^(.) *p*(*N*,./*B*, *t* ^2^) *δ*(.) *e*(*T*) *φ*(*t*) *α*(*t* ^2^) *c*(*T*)	22	0.000	0.699	1.1
*S*(.) *Ψ*(*s*,.) *π* ^*B*^(.) *ω* ^*B*^(.) *p*(*N*,./*B*, *t* ^2^) *δ*(.) *e*(*T*) *φ*(*t*) *α*(*t* ^2^) *c*(*t* ^2^)	23	2.048	0.251	1.1
*S*(.) *Ψ*(*s*,.) *π* ^*B*^(.) *ω* ^*B*^(.) *p*(*N*,./*B*, *t* ^2^) *δ*(.) *e*(*T*) *φ*(*t*) *α*(*t* ^2^) *c*(*t*)	26	5.369	0.048	1.1
*S*(*s*) *Ψ*(*s*,.) *π* ^*B*^(.) *ω* ^*B*^(.) *p*(*N*,./*B*, *t* ^2^) *δ*(.) *e*(*T*) *φ*(*t*) *α*(*t* ^2^) *c*(*T*)	24	11.581	0.002	1.1
*S*(.) *Ψ*(*s*,.) *π* ^*B*^(.) *ω* ^*B*^(.) *p*(*N*,./*B*, *t* ^2^) *δ*(.) *e*(*T*) *φ*(*t*) *α*(*T*) *c*(*T*)	21	75.26	0.000	1.1
Burray known reproductive females				
*S*(.) *Ψ*(*s*,.) *π* ^*B*^(.) *ω* ^*B*^(*T*) *p*(*s*, *t* ^2^) *δ*(.) *e*(*T*) *φ*(*T*) *α*(*t* ^2^) *c*(*T*)	21	0.000	0.384	1.486
*S*(.) *Ψ*(*s*,.) *π* ^*B*^(.) *ω* ^*B*^(*t*) *p*(*s*, *t* ^2^) *δ*(.) *e*(*T*) *φ*(*T*) *α*(*t* ^2^) *c*(*T*)	25	0.600	0.285	1.486
*S*(.) *Ψ*(*s*,.) *π* ^*B*^(.) *ω* ^*B*^(.) *p*(*s*, *t* ^2^) *δ*(.) *e*(*T*) *φ*(*T*) *α*(*t* ^2^) *c*(*T*)	20	0.629	0.281	1.486
*S*(.) *Ψ*(*s*,.) *π* ^*B*^(.) *ω* ^*B*^(.) *p*(*s*, *t* ^2^) *δ*(.) *e*(*T*) *φ*(*T*) *α*(*t* ^2^) *c*(*t* ^2^)	24	4.267	0.045	1.486
*S*(.) *Ψ*(*s*,.) *π* ^*B*^(.) *ω* ^*B*^(.) *p*(*s*, *t* ^2^) *δ*(.) *e*(*T*) *φ*(*T*) *α*(*t* ^2^) *c*(*t*)	27	8.631	0.005	1.486
Dunvegan ≥ 4‐year old females				
*S*(*T*) *Ψ*(*s*,.) *π* ^*B*^(.) *ω* ^*B*^(*T*) *p*(*N*,./*B*, *t* ^2^) *δ*(.) *e*(*T*) *φ*(*t* ^2^) *α*(*t* ^2^) *c*(*T*)	22	0.000	0.442	1.726
*S*(*T*) *Ψ*(*s*,.) *π* ^*B*^(.) *ω* ^*B*^(.) *p*(*N*,./*B*, *t* ^2^) *δ*(.) *e*(*T*) *φ*(*t* ^2^) *α*(*t* ^2^) *c*(*T*)	21	0.828	0.292	1.726
*S*(*T*) *Ψ*(*s*,.) *π* ^*B*^(.) *ω* ^*B*^(*t*) *p*(*N*,./*B*, *t* ^2^) *δ*(.) *e*(*T*) *φ*(*t* ^2^) *α*(*t* ^2^) *c*(*T*)	26	1.015	0.266	1.726
*S*(*T*) *Ψ*(*s*,.) *π* ^*B*^(.) *ω* ^*B*^(*T*) *p*(*N*,./*B*, *t* ^2^) *δ*(.) *e*(*t* ^2^) *φ*(*s*,*t* ^2^) *α*(*T*) *c*(*T*)	20	23.14	0.000	1.726
*S*(*T*) *Ψ*(*s*,.) *π* ^*B*^(.) *ω* ^*B*^(*T*) *p*(*N*,./*B*, *t* ^2^) *δ*(.) *e*(*s*,*t* ^2^) *φ*(*T*) *α*(*T*) *c*(*T*)	22	25.32	0.000	1.726
Dunvegan known reproductive females				
*S*(*T*) *Ψ*(*s*,.) *π* ^*B*^(.) *ω* ^*B*^(*T*) *p*(*N*,*t*/*B*, *t* ^2^) *δ*(.) *e*(*t* ^2^) *φ*(*t* ^2^) *α*(*t* ^2^) *c*(*T*)	24	0.000	0.784	0.88
*S*(*T*) *Ψ*(*s*,.) *π* ^*B*^(.) *ω* ^*B*^(.) *p*(*N*,*t*/*B*, *t* ^2^) *δ*(.) *e*(*t* ^2^) *φ*(*t* ^2^) *α*(*t* ^2^) *c*(*T*)	23	2.591	0.215	0.88
*S*(*T*) *Ψ*(*s*,.) *π* ^*B*^(.) *ω* ^*B*^(*T*) *p*(*N*,*t*/*B*, *t* ^2^) *δ*(.) *e*(*t* ^2^) *φ*(*t* ^2^) *α*(*T*) *c*(*T*)	23	13.652	0.001	0.88
*S*(*T*) *Ψ*(*s*,.) *π* ^*B*^(.) *ω* ^*B*^(*T*) *p*(*N*,*t*/*B*, *t* ^2^) *δ*(.) *e*(*t* ^2^) *φ*(*T*) *α*(*T*) *c*(*T*)	22	25.862	0.000	0.88
*S*(*T*) *Ψ*(*s*,.) *π* ^*B*^(.) *ω* ^*B*^(*T*) *p*(*N*,*t*/*B*, *t* ^2^) *δ*(.) *e*(*T*) *φ*(*T*) *α*(*T*) *c*(*T*)	21	26.570	0.000	0.88
Loch Fleet ≥ 4‐year old females				
*S*(*s*, *T*) *Ψ*(*s*,.) *π* ^*B*^(.) *ω* ^*B*^(.) *p*(*N*,*T*/*B*, *t* ^2^) *δ*(.) *e*(*s*,*T*) *φ*(*T*) *α*(*t* ^2^) *c*(*t* ^2^)	24	0.000	1.000	1.646
*S*(*s*, *T*) *Ψ*(*s*,.) *π* ^*B*^(.) *ω* ^*B*^(.) *p*(*N*,*T*/*B*, *t* ^2^) *δ*(.) *e*(*s*,*T*) *φ*(*T*) *α*(*t* ^2^) *c*(*T*)	23	37.495	0.000	1.646
*S*(*s*, *T*) *Ψ*(*s*,.) *π* ^*B*^(.) *ω* ^*B*^(*T*) *p*(*N*,*T*/*B*, *t* ^2^) *δ*(.) *e*(*s*,*T*) *φ*(*T*) *α*(*t* ^2^) *c*(*t* ^2^)	25	40.518	0.000	1.646
*S*(*s*, *T*) *Ψ*(*s*,.) *π* ^*B*^(.) *ω* ^*B*^(*t*) *p*(*N*,*T*/*B*, *t* ^2^) *δ*(.) *e*(*s*,*T*) *φ*(*T*) *α*(*t* ^2^) *c*(*t* ^2^)	39	103.119	0.000	1.646
*S*(*s*, *T*) *Ψ*(*s*,.) *π* ^*B*^(.) *ω* ^*B*^(.) *p*(*N*,*T*/*B*, *t* ^2^) *δ*(.) *e*(*s*,*T*) *φ*(*T*) *α*(*T*) *c*(*T*)	22	193.958	0.000	1.646
Loch Fleet known reproductive females				
*S*(*s*, *T*) *Ψ*(*s*,.) *π* ^*B*^(.) *ω* ^*B*^(.) *p*(*N*,./*B*, *t* ^2^) *δ*(.) *e*(*s*,*t* ^2^) *φ*(*t* ^2^) *α*(*t*) *c*(*t*)	32	0.000	0.997	0.832
*S*(*s*, *T*) *Ψ*(*s*,.) *π* ^*B*^(.) *ω* ^*B*^(.) *p*(*N*,./*B*, *t* ^2^) *δ*(.) *e*(*s*,*t* ^2^) *φ*(*t* ^2^) *α*(*t*) *c*(*T*)	27	11.956	0.002	0.832
*S*(*s*, *T*) *Ψ*(*s*,.) *π* ^*B*^(.) *ω* ^*B*^(*T*) *p*(*N*,./*B*, *t* ^2^) *δ*(.) *e*(*s*,*t* ^2^) *φ*(*t* ^2^) *α*(*t*) *c*(*t*)	33	19.162	0.000	0.832
*S*(*s*, *T*) *Ψ*(*s*,.) *π* ^*B*^(.) *ω* ^*B*^(.) *p*(*N*,./*B*, *t* ^2^) *δ*(.) *e*(*s*,*t* ^2^) *φ*(*t*) *α*(*T*) *c*(*T*)	25	292.385	0.000	0.832
*S*(*s*, *T*) *Ψ*(*s*,.) *π* ^*B*^(.) *ω* ^*B*^(.) *p*(*N*,./*B*, *t* ^2^) *δ*(.) *e*(*s*,*t* ^2^) *φ*(*T*) *α*(*T*) *c*(*T*)	21	335.282	0.000	0.832

*Note:* Parameters are fitted as constant (.), varying between breeder and non‐breeder (s), time‐dependent (year or week within years, t) or with a linear (*T*) or quadratic (*t* ^2^) trend. The inflation factor (*ĉ*) was applied to correct model selection when *ĉ* > 1. Only the top 5 models for each analysis are shown, to capture those models with AICc or QAICc weights > 0.

At Dunvegan, 232 adult females were identified, of which 169 pupped at least once. GOF tests showed a small lack of fit (*χ* ^2^ = 82.871, df = 48, *p* = 0.001) of the ≥ 4 years old female dataset, with some over‐dispersion (*ĉ* = 1.726) and presence of transience (test3Gsr) for breeders on year (2018) only. Model selection and parameter associated variance were corrected by *ĉ*. Three models fell within 2 QAICc of the top model (Table 3), with fecundity rate modelled as constant (0.728, 95% CI: 0.646–0.796), varying by year (range 0.613 to 0.901), and with a linear trend (significant negative trend based on β‐parameter values, with estimates ranging from 0.792 to 0.622). Model‐averaged probabilities of pupping were 0.215 (95% CI: 0.076–0.477) after a year with no pup (*Ψ* ^*NB*^) and 0.910 (95% CI: 0.784–0.966) following a year with pup (*Ψ* ^*BB*^). Data from known reproductive females had a good fit (*χ* ^2^ = 33.49, df = 38, *p* = 0.678) and *ĉ* = 0.88. The most supported model estimated a fecundity rate with a negative time trend from 0.921 to 0.758. A model with constant fecundity rate (0.883, 95% CI: 0.823–0.924) was also supported (2.59 ΔAICc). Model‐averaged probabilities of pupping were 0.695 (95% CI: 0.437–0.870) after a year with no pup (*Ψ* ^*NB*^) and 0.714 (95% CI: 0.616–0.795) following a year with pup (*Ψ* ^*BB*^) (Table 2).

At Loch Fleet, 153 adult females were identified, of which 135 pupped at least once. The ≥ 4 ‐years old female dataset had a small lack of fit (*χ* ^2^ = 65.852, df = 40, *p* = 0.006) and some over‐dispersion (*ĉ* = 1.646). The best model had a constant fecundity rate, and models with time‐dependent or trend on fecundity rate did not receive support. Because the first 4 years of data (2006 to 2009) included only known reproductive females, the resulting fecundity rate was biased upwards (e.g., *ω* ^B^_2006_≈1). Instead, the most supported model was fitted to data from 2010 onwards, estimating a fecundity rate of 0.769 (95% CI: 0.725–0.807), a probability of pupping of 0.360 (95% CI: 0.219–0.530) after a year with no pup (*Ψ* ^*NB*^) and of 0.895 (95% CI: 0.818–0.941) following a year with pup (*Ψ* ^*BB*^). Data from known reproductive females (2006–2021) had an overall good fit (*χ* ^2^ = 25.964, df = 31, *p* = 0.728) and *ĉ* = 0.832. Only one model received support (ΔAICc = 11.95), which had a constant fecundity rate (0.872, 95% CI: 0.847–0.894), a probability of pupping of 0.607 (95% CI: 0.490–0.713) after a year with no pup (*Ψ* ^*NB*^) and of 0.883 (95% CI: 0.850–0.910) following a year with pup (*Ψ* ^*BB*^) (Table 2).

**FIGURE 3:**
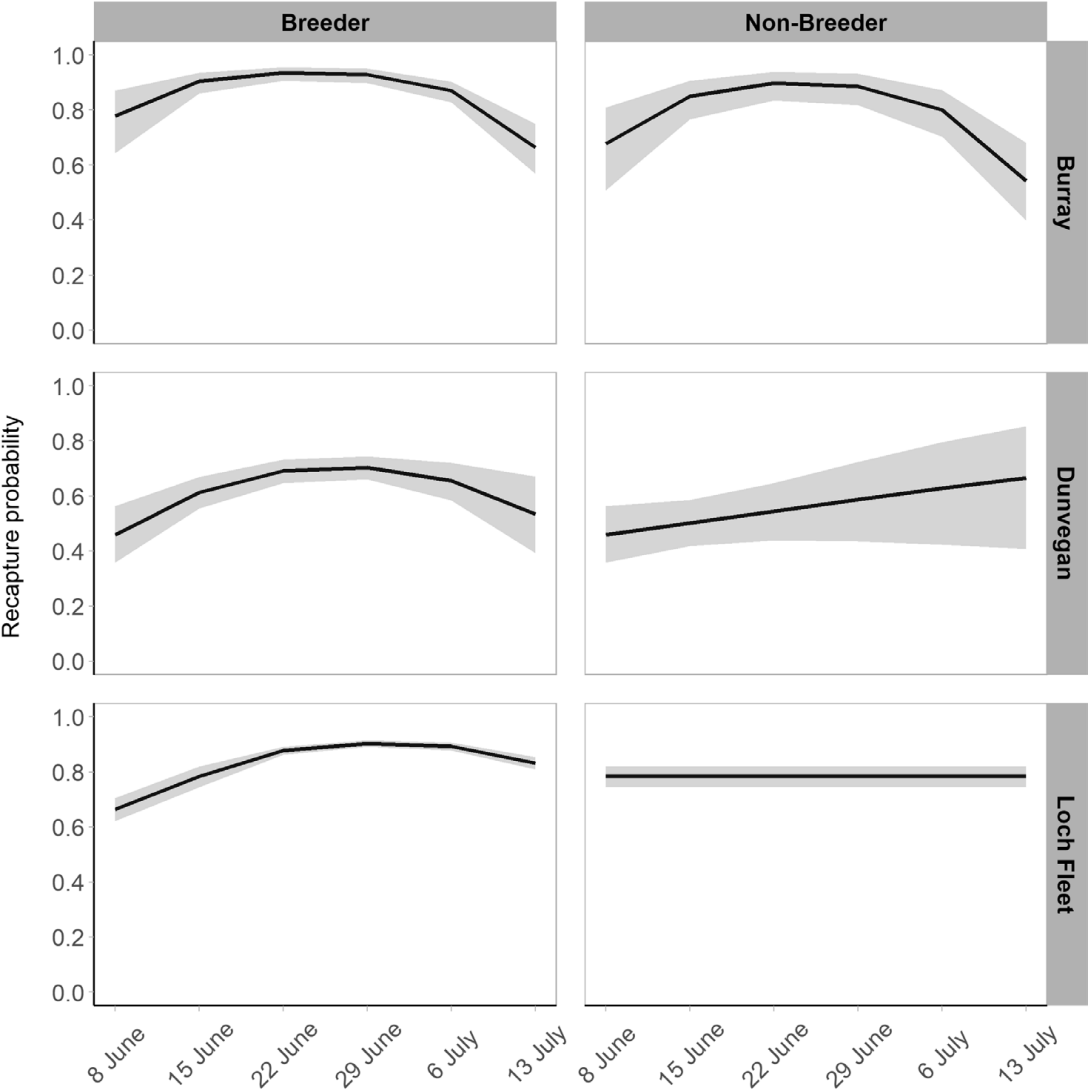
Recapture probability (*p*) estimates for each study area with 95% confidence intervals (in grey) from the dataset of known reproductive females. Estimates for Burray and Dunvegan are model‐averaged over the most supported models. Estimates for Loch Fleet are from the single most supported model.

**FIGURE 4:**
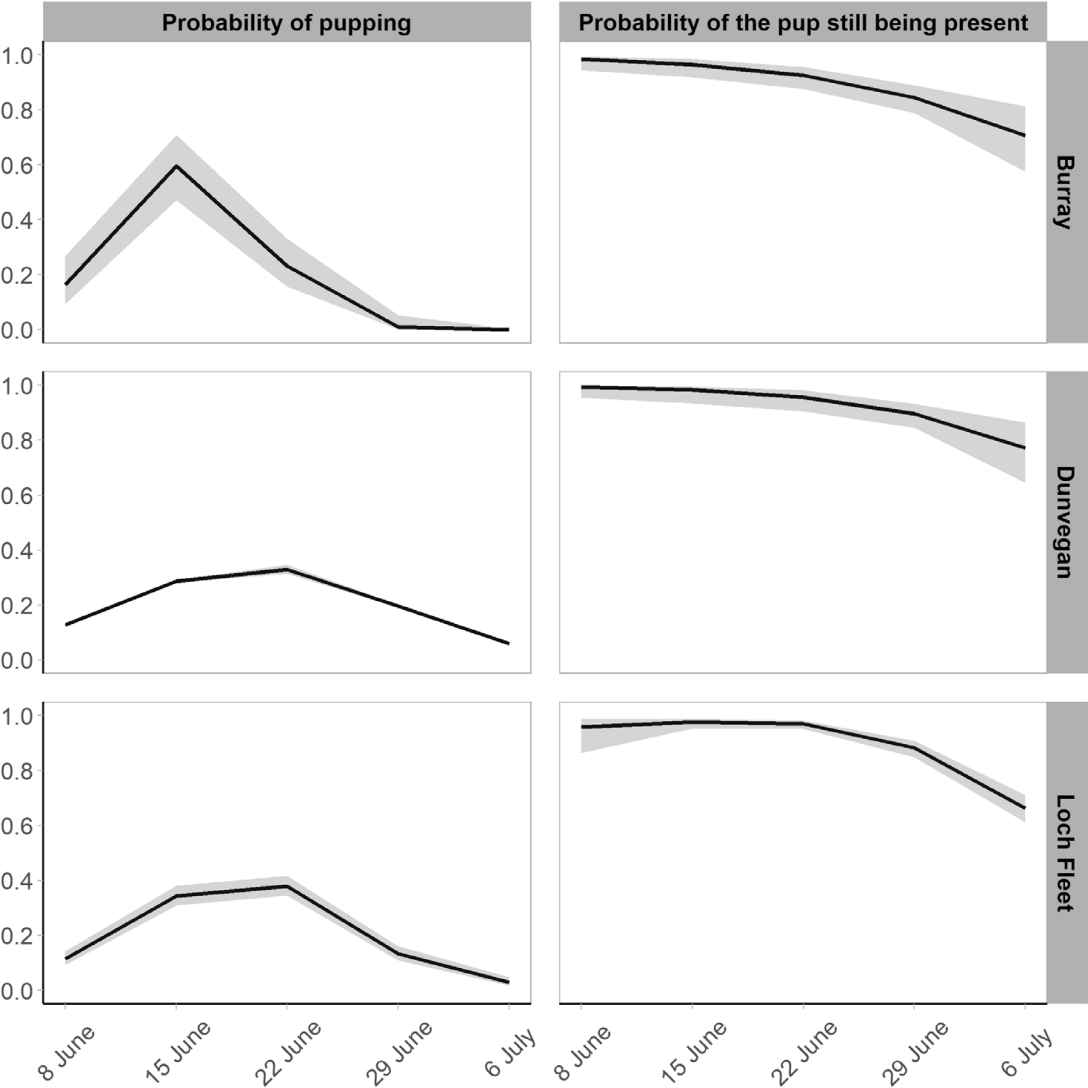
Estimates of the probability of pupping (α) and the probability of the pup still being present (c) for each study area with 95% confidence intervals (in grey) from the dataset of known reproductive females. Estimates for Burray and Dunvegan are model‐averaged over the most supported models. Estimates for Loch Fleet are from the single most supported model.

Female state‐dependent survival was not supported for Burray or Dunvegan datasets but received support for Loch Fleet, with lower survival for non‐breeders than breeders in both datasets. Recapture probabilities (*p*) and the probabilities of pupping (*α*) and of the pup still being present (*c*) are plotted in Figures 3 and 4 for all three sites for known reproductive females. Recapture probabilities were high for breeders and non‐breeders (see Table 2, Figure 3). The probability of pupping (*α*) was greatest around mid‐June for Burray and slightly later for Dunvegan and Loch Fleet. The probability of the pup still being present (*c*) declined at all sites by the end of June coinciding with weaning of the pups (Figure 4).

## 4 Discussion

In this study we estimated apparent survival rates and fecundity rates of harbour seals using mark–recapture models applied to photo‐ID data collected in areas with contrasting population trajectories in Scotland. The results suggest that survival is the main factor driving differences in population trajectories, with reduced apparent adult survival in areas of decline. Analysis of fecundity rates showed a similar overall pattern across sites, although the contrasts in fecundity rate were less pronounced than for adult survival.

### 4.1 Differences in Adult Survival

Burray had the lowest apparent adult survival rates. This site, within the North Coast and Orkney SMU, has declined by 85% since its highest estimated abundance in 2002 (Russell et al. 2024) and was still declining at an annual rate of 7%–10% (Hall et al. 2024) at the time of this study. In contrast, Loch Fleet (within the Moray Firth SMU, an area where abundance has shown no clear trend since 2003) had the highest overall and sex‐specific adult apparent survival rates. These estimates were very similar to previous estimates from this population using a shorter time series (2006–2011; Cordes and Thompson 2014). At Dunvegan (within West Scotland SMU showing stable/increasing trends at the time of this study), apparent survival estimates were also high for all adults combined but lower when sex‐specific models were run. Because seals were aged from photographs at Burray and Dunvegan, capture histories might have included some juveniles. Assuming survival of immature seals is lower than that of adults (Hastings et al. 2012), Loch Fleet survival might be slightly biased towards older seals (especially for males), which could account for some of the differences in survival rates between sites. However, efforts were made to minimise the inclusion of juvenile seals in the analysis by having the same experienced researcher involved in ageing all individual seals for Burray and Dunvegan and by using a conservative approach when classifying (i.e., animals were classed as juvenile if in disagreement and did not enter the analysis in that year).

Mark–recapture apparent survival probabilities represent a combination of true survival and site fidelity, where permanent emigration cannot be distinguished from death and will underestimate survival. Harbour seals show sex‐ and age‐specific variation in haulout usage and movements (Härkönen et al. 1999; Lowry et al. 2001; Thompson et al. 1997), as well as seasonal switches in haulout site usage (e.g., Thompson, Miller, et al. 1994). Long‐distance movements have been documented, especially for younger animals (Thompson, Kovacs, and McCocConnell 1994; Greig 2011). However, harbour seals display high natal site fidelity, especially in adult females, although reduced fidelity to natal site has been seen in males with age (Härkönen and Harding 2001). Emigration is not considered a major contributing factor to the declines in Scotland (Carroll et al. 2020; McConnell et al. 2017; Olsen et al. 2017; Russell et al. 2015; SCOS 2020), and photo‐ID and telemetry data collected during the study period (Arso Civil et al. 2018 and this study) suggest permanent emigration is unlikely to have a significant effect on apparent survival estimates.

Compared with published estimates, apparent survival rates from Burray, Dunvegan and Loch Fleet all fall within the reported range for harbour seals. For example, data from dead seals in the Kattegat‐Skagerrak region after the 1988 epizootic estimated female survival of 0.95 and male survival of 0.91 under an assumed population increase rate of 0.11 (Härkönen and Heide‐Jørgensen 1990) and Svensson (2012) reports female adult survival of 0.97 based on parameterisation of data from this same area. In the Gulf of Alaska, Pitcher and Calkins (1979) report female survival of 0.89 and male survival of 0.87 in the 1970s. Bigg (1969) reports some of the lowest estimates of survival (0.71 for > 5‐year old males and 0.85 for > 5‐year old females) based on the age structure of samples of dead seals in British Columbia during an assumed period of population stability. The only other photo‐ID based survival estimate from outside Scotland is from Tugidak Island (Alaska), where Hastings et al. (2012) estimated female survival of 0.929 (95% CI 0.858–0.966) and male survival of 0.879 (95% CI 0.784–0.936) after a recent period of population increase. The reported survival rates from these other harbour seal populations, which are mostly estimated during periods of population stability or increase, are closer to the higher estimates from Loch Fleet and Dunvegan, while Burray estimates were lower than most. While the reduced survival estimated for Burray might not completely account for the wider North Coast and Orkney SMU declining trend, it provides evidence that adult survival is reduced in this area of decline.

### 4.2 Resighting Probabilities

Annual recapture rates from the survival models were generally high in Burray (0.887) and Loch Fleet (0.797 to 0.884), but lower at Dunvegan (0.341 to 0.617). The few other photo‐ID studies report similar recapture probabilities (0.56, Hastings et al. 2012; 0.62, Mackey et al. 2008; 0.97, Cordes and Thompson 2014). Comparatively lower recapture probabilities at Dunvegan could be reflective of a lower level of site fidelity by individual seals or a higher presence of transient animals, although a telemetry study in 2004/05 in that area showed that while seals occasionally travelled long distances, individuals showed a relatively high degree of site fidelity, particularly on a short temporal scale (i.e., several months) (Cunningham et al. 2009). The number of animals counted and identified at Dunvegan declined over the study period (Table 1). Interestingly, we also observed a significant reduction in the number of juveniles photo identified at Dunvegan. Similar changes in population age structure were observed in Orkney in the late 1990s before the population decline was detected, with a marked reduction in abundance of seals and an absence of yearlings in particular (Thompson et al. 2001). However, the wider West Scotland—Central SMU population trend was increasing at the time this study was conducted (Russell et al. 2024), and estimated adult apparent survival was high here, suggesting the decrease in counts and photo‐identified seals may be due to movement rather than increased mortality. Harbour seals, including females with pups, may have used nearby alternative sites (Cunningham et al. 2009; Thompson, Kovacs, and McCocConnell 1994; Thompson, Miller, et al. 1994) outside the photo‐ID area. Boat operators at Dunvegan have observed increased cases of male grey seals harassing and predating on harbour seal pups since 2019 (Raymond Coughlin, pers. comm). Predation by adult male North Atlantic grey seals on both grey and harbour seals is reported in different populations (Bishop et al. 2016; van Neer et al. 2015) and has been documented in all three SMUs included in this study (Langley 2024; Langley et al. 2025). The localised nature at Dunvegan however might make it a significant factor contributing to displacement of animals in this area. More generally, data on movement patterns of animals within and between seasons and on long‐term site fidelity is needed to understand how much of the survival and recapture probabilities reflect temporary/long‐term dispersal or mortality. It also highlights the importance of long‐term time series and incorporating data on population structure alongside abundance into population dynamics studies to understand early signs of potential declines (e.g., Carroll et al. 2025).

### 4.3 Reproductive Rates

As expected, fecundity rates (i.e., unconditional reproductive rates representing the proportion of females with a pup in each year) were higher for known reproductive females compared to the adult female dataset which included younger, sexually mature but non‐reproductive females. Fecundity rates at Loch Fleet were similar to those estimated in Cordes and Thompson (2014) for this population using a shorter time series. Dunvegan had a similarly high fecundity rate, while Burray had a slightly lower fecundity rate, although the 95% confidence intervals overlapped between areas. The estimated fecundity rates here are all slightly lower in this stidy (0.809–0.883) than those reported for harbour seals in other populations, where typically > 90% of adult females are reported pregnant (e.g., Bjørge 1992; Boulva and McLaren 1979; Härkönen and Heide‐Jørgensen 1990; Lydersen and Kovacs 2005). These other studies were mostly based on examination of sampled ovaries or evidence of foetus or lactation in harvested females. However, estimation of reproductive rates counting placental scars, corpus luteum, and corpus albicans may result in overestimated rates (Cordes and Thompson 2014), which might account for some of the differences with the photo‐ID based studies. Lydersen and Kovacs (2005) on the other hand, derived proportion of females pregnant or with pup from a live‐capture study, which might have resulted in a slightly higher fecundity rate than just based on association with a pup. Finally, a study using blood and blubber samples from harbour seals captured and released between 2015 and 2018 at the study sites of Burray, Dunvegan and Loch Fleet estimated pregnancy rates from progesterone concentrations (Hall et al. 2020). Their results indicated > 60% of mature females were categorised as pregnant in all three areas, but proportions changed depending on whether plasma, blubber or a combined diagnostic was used. The latter resulted in higher and more accurate pregnancy rates of 80%–100% mature females classified as pregnant. These results, which were available before the photo‐ID analysis was finalised, suggested the decline in harbour seal numbers was unlikely to be driven by changes in pregnancy proportions, although the small sample sizes and wide confidence intervals did not permit robust conclusions at the time.

At Dunvegan and Burray, models with a (significant) declining trend in fecundity over the 6‐years study also received some support through model selection (within 3 AICc from the top model), alongside models without a fitted trend. At Dunvegan, the photo‐ID data showed an increase in the proportion of adult females seen initially pregnant that were then not seen later in the season (i.e., a pup could not be confirmed as females were not seen). This can only be explained if pregnant females left the study site before pupping or pups died or were separated before the mum‐pup pair could be observed and recorded. In either case this could result in an apparent decline in fecundity rates over time, as females would enter the capture histories as not seen with a pup (unknown state) but leave before the pup was recorded. In the case of Burray, photo‐ID data did not show the same evidence regarding pregnant females potentially moving away from the study site and thus it is possible that the support for a model with a declining fecundity might not be due to movements in this case. However, for both sites, models without a trend in fecundity were also supported and thus longer‐term data would be needed to investigate this further to understand if a decline in fecundity is supported by the data.

Accounting for misclassification or uncertainty in breeding status from photo‐ID observations is important to correct fecundity (breeding) probabilities (Cheney et al. 2019; Kendall et al. 2003), as pups might not always be seen with a female (e.g., the pup is not photographed, obscured by the female, separated from the female or dead). The fecundity models used here indicated there was > 80% probability of correctly classifying breeders at all three sites. Allo‐suckling (i.e., nursing another's young) was observed at Burray every year of the study and at higher rates than those reported in other phocid populations (see Arso Civil et al. 2021). However, allo‐suckling should not have affected the probability of correctly classifying a breeder (and consequently biasing fecundity rates) given that within this modelling framework, a female suckling their own pup or another pup would still enter the capture history as a breeder. Also, most allo‐suckling events occurred 14–24 days after a female gave birth, rather than in the first 2 weeks after birth (Arso Civil et al. 2021) and thus females would have most likely been recorded with their pup by the time allo‐suckling occurred. The modelling framework used here also estimates conditional reproductive rates, that is conditional on the female's state in the previous year, *Ψ* ^BB^ and *Ψ* ^NB^, representing the probabilities of pupping after a year with no pup or following a year with a pup, respectively. At Loch Fleet, *Ψ* ^BB^ and *Ψ* ^NB^ were similar to the estimates in Cordes and Thompson (2014) for this population. For known reproductive females, the probability of pupping following a year with a pup was lower in Burray and Dunvegan compared to Loch Fleet, while the probability of pupping after a year with no pup was similar across sites. However, conditional reproductive rates had generally wide 95% CI in both ≥ 4 years old and known reproductive females, which limited comparisons between sites.

Photo‐ID data was collected at breeding sites, which may be favoured by mature females given sex and age preferences, or segregation at haulout sites (Härkönen et al. 1999; Härkönen and Harding 2001; Cordes and Thompson 2015). If non‐breeding females were absent from breeding sites, it might overestimate recruitment rate for the population as a whole. However, both breeding and (assumed) non‐breeding (i.e., not seen with a pup) females were photographed at all sites, and recapture probabilities for both groups were high (see Table 2). Telemetry data in 2016 and 2017 at Burray also indicated that both breeders and non‐breeders were using the haulout site throughout the season (Arso Civil et al. 2018). Cordes and Thompson (2015) showed how, in Loch Fleet, individual seals used the haulout site to the same extent throughout the year, with changes in counts likely reflecting changes in resighting rates (peaking during the breeding season) rather than changes in abundance. Thus, we believe a bias in fecundity rates from sampling at breeding sites is unlikely in this study.

## 5 Conclusions and Wider Implications

This study generated concurrent estimates of adult survival and fecundity rates for harbour seals at sites of contrasting population trajectories, showing that the extrinsic and/or intrinsic drivers at declining sites are likely acting on adult survival, while support for fecundity driving the changes was not as strong. The results fill in a recognised data gap to understand the drivers behind different population dynamics in Scotland and provide essential information for future parameterisation of population assessments to explore impact of human activities and inform management decisions.

Individual‐based studies like this study help understand the dynamics behind changes in population size (e.g., Gaillard et al. 1998, Fritz et al. 2014; Arso Civil et al. 2018), which can help narrow down the causal factors (e.g., changes in prey, competition, predation, disease, climate pathways) potentially affecting vital rates (e.g., Pendleton et al. 2006; Fay et al. 2017; Hastings et al. 2023; Herrera Fuchs et al. 2025). For example, predation and interspecific competition has been linked to dramatic declines in harbour seals on Sable Island (Canada), affecting all age classes (Bowen et al. 2003). Wider‐scale, ecosystem‐level changes in Alaska and the Bering Sea have been linked to declines in several marine mammal populations including harbour seals, northern fur seals and Steller sea lions (Hastings et al. 2012; Jemison and Kelly 2001). In Scotland, potential causes of the declines include competition and predation by grey seals, predation by killer whales and exposure to toxins from harmful algae blooms (Hall et al. 2024, Langley 2024; Langley et al. 2025; Sutherland 2024). The conclusion here that adult survival is reduced in areas of decline can help direct future efforts to understand how and to what level each of the likely causes of decline is acting in different areas, and whether they are doing so in isolation or in combination with other factors.

Previous work used state‐space models incorporating harbour seal age‐structured dynamics and count data from aerial surveys to investigate proximate causes and drivers affecting seal population trends in the Moray Firth (Matthiopoulos et al. 2014; Caillat et al. 2019). Photo‐ID based estimates of apparent survival and fecundity from this study can now be used to further develop that modelling framework to include empirical data from different areas with contrasting trajectories and investigate hypothesis for trends in vital rates (Jacobson et al., n.d. in prep). These stage‐structured population dynamics models can be used to investigate if the hypothesised mechanisms for decline (i.e., reduction in survival rate) are supported by the aerial survey count data and explore potential drivers influencing vital rates. More widely, our study provides essential information to parameterise future population assessments for harbour seals, for example through population viability analysis (PVA; Lacy 2000) or other age‐structured population models to explore the impact of anthropogenic pressures and environmental changes on populations and to guide management decisions and future research.

## Author Contributions

**M. Arso Civil:** conceptualization (lead), data curation (lead), formal analysis (lead), funding acquisition (supporting), investigation (equal), methodology (lead), project administration (equal), supervision (supporting), writing – original draft (lead), writing – review and editing (equal). **S. Tapp:** data curation (supporting), writing – review and editing (supporting). **J. Dickens:** data curation (supporting), writing – review and editing (supporting). **I. Langley:** data curation (supporting), writing – original draft (supporting), writing – review and editing (supporting). **H. M. Hiley:** data curation (supporting), writing – review and editing (supporting). **M. Terrapon:** data curation (supporting), writing – review and editing (supporting). **E. Hague:** data curation (supporting), writing – review and editing (supporting). **R. C. Hewitt:** data curation (supporting), methodology (supporting), writing – original draft (supporting), writing – review and editing (supporting). **L. S. Cordes:** data curation (supporting), formal analysis (supporting), writing – original draft (supporting), writing – review and editing (supporting). **I. M. Graham:** conceptualization (supporting), data curation (supporting), writing – original draft (supporting), writing – review and editing (supporting). **B. J. Cheney:** conceptualization (supporting), formal analysis (supporting), writing – original draft (supporting), writing – review and editing (supporting). **P. M. Thompson:** conceptualization (supporting), funding acquisition (supporting), writing – original draft (supporting), writing – review and editing (supporting). **A. Hall:** conceptualization (supporting), funding acquisition (lead), project administration (equal), supervision (supporting), writing – original draft (supporting), writing – review and editing (supporting). **C. E. Sparling:** conceptualization (supporting), funding acquisition (equal), project administration (equal), supervision (supporting), writing – original draft (supporting), writing – review and editing (supporting).

## Conflicts of Interest

The authors declare no conflicts of interest.

## Data Availability

The research data and R‐code underpinning this publication can be accessed at https://doi.org/10.17630/bd7fe8db‐10e3‐45ee‐b067‐19c9182f23e5, and should be cited as Arso Civil, M., Tapp, S., Dickens, J., Langley, I., Hiley, H.M., Terrapon, M., Hague, E., Hewitt, R.C., Cordes, L.S., Graham, I.M., Cheney, B.J., Thompson, P.M., Hall, A.J., and Sparling, C.E. (2025) Reduced adult survival estimated in areas of decline of harbour seal populations in Scotland (dataset and R ‐code). Dataset. University of St Andrews Research Portal. https://doi.org/10.17630/bd7fe8db‐10e3‐45ee‐b067‐19c9182f23e5.

## References

[ece372349-bib-0120] Anderson, D. R. , K. P. Burnham , and G. C. White . 1994. “AIC Model Selection in Overdispersed Capture‐Recapture Data.” Ecology 75, no. 6: 1780–1793. 10.2307/1939637.

[ece372349-bib-0002] Arso Civil, M. , E. Hague , I. Langley , and L. Scott‐Hayward . 2021. “Allo‐Suckling Occurrence and Its Effect on Lactation and Nursing Duration in Harbour Seals (*Phoca vitulina*) in Orkney, Scotland.” Behavioral Ecology and Sociobiology 75, no. 8: 121. 10.1007/s00265-021-03051-y.

[ece372349-bib-0004] Arso Civil, M. , S. Smout , J. Onoufriou , et al. 2016. Harbour Seal Decline—Vital Rates and Drivers. Report to Scottish Government HSD2. http://www.smru.st‐andrews.ac.uk/files/2016/10/HSD‐2‐annual‐report‐year‐1.pdf.

[ece372349-bib-0117] Arso Civil, M. , S. C. Smout , C. Duck , et al. 2018. “Harbour Seal Decline – Vital Rates and Drivers.” Report to Scottish Government HSD2.

[ece372349-bib-0007] Bigg, M. A. 1969. The Harbour Seal in British Columbia. Vol. 172, 1–33. Fisheries Research Board of Canada Bulletin.

[ece372349-bib-0008] Bishop, A. M. , J. Onoufriou , S. Moss , P. P. Pomeroy , and S. D. Twiss . 2016. “Cannibalism by a Male Grey Seal ( *Halichoerus grypus* ) in the North Sea.” Aquatic Mammals 42, no. 2: 137–143.

[ece372349-bib-0009] Bjørge, A. 1992. “The Reproductive Biology of the Harbour Seal, *Phoca vitulina* L., in Norwegian Waters.” Sarsia 77, no. 1: 47–51. 10.1080/00364827.1992.10413491.

[ece372349-bib-0010] Blanchet, M.‐A. , C. Vincent , J. N. Womble , S. M. Steingass , and G. Desportes . 2021. “Harbour Seals: Population Structure, Status, and Threats in a Rapidly Changing Environment.” Oceans 2: 41–63.

[ece372349-bib-0011] Bolger, D. T. , T. A. Morrison , B. Vance , D. Lee , and H. Farid . 2012. “A Computer‐Assisted System for Photographic Mark–Recapture Analysis.” Methods in Ecology and Evolution 3, no. 5: 813–822. 10.1111/j.2041-210X.2012.00212.x.

[ece372349-bib-0012] Boness, D. J. , W. D. Bowen , and O. T. Oftedal . 1994. “Evidence of a Maternal Foraging Cycle Resembling That of Otariid Seals in a Small Phocid, the Harbor Seal.” Behavioral Ecology and Sociobiology 34, no. 2: 95–104.

[ece372349-bib-0013] Boulva, J. , and I. A. McLaren . 1979. Biology of the Harbor Seal, *Phoca vitulina* , in Eastern Canada. Dept. of Fisheries and Oceans.

[ece372349-bib-0014] Bowen, W. D. , S. L. Ellis , S. J. Iverson , and D. J. Boness . 2003. “Maternal and Newborn Life‐History Traits During Periods of Contrasting Population Trends: Implications for Explaining the Decline of Harbour Seals (*Phoca vitulina*), on Sable Island.” Journal of Zoology 261: 155–163. 10.1017/s0952836903004047.

[ece372349-bib-0016] Brownlow, A. , J. Onoufriou , A. Bishop , N. Davison , and D. Thompson . 2016. “Corkscrew Seals: Grey Seal ( *Halichoerus grypus* ) Infanticide and Cannibalism May Indicate the Cause of Spiral Lacerations in Seals.” PLoS One 11: e0156464. 10.1371/journal.pone.0156464. 27254025 PMC4890781

[ece372349-bib-0017] Burnham, K. P. , and D. R. Anderson . 2002. Model Selection and Inference: A Practical Information Theoretic Approach (2nd ed.). Springer.

[ece372349-bib-0018] Caillat, M. , L. Cordes , P. M. Thompson , J. Matthiopoulos , and S. Smout . 2019. “Use of State‐Space Modelling to Identify Ecological Covariates Associated With Trends in Pinniped Demography.” Aquatic Conservation: Marine and Freshwater Ecosystems 29, no. S1: 101–118.

[ece372349-bib-0019] Carroll, D. , E. Infantes , E. V. Pagan , and K. C. Harding . 2025. “Approaching a Population‐Level Assessment of Body Size in Pinnipeds Using Drones, an Early Warning of Environmental Degradation.” Remote Sensing in Ecology and Conservation 11: 156–171. 10.1002/rse2.413.

[ece372349-bib-0020] Carroll, E. L. , A. Hall , M. T. Olsen , A. B. Onoufriou , O. E. Gaggiotti , and D. J. F. Russell . 2020. “Perturbation Drives Changing Metapopulation Dynamics in a Top Marine Predator.” Proceedings of the Royal Society B 287: 20200318. 10.1098/rspb.2020.0318. 32486973 PMC7341938

[ece372349-bib-0021] Carter, M. I. D. , L. Boehme , M. A. Cronin , et al. 2022. “Sympatric Seals, Satellite Tracking and Protected Areas: Habitat‐Based Distribution Estimates for Conservation and Management.” Frontiers in Marine Science 9: 875869. 10.3389/fmars.2022.875869.

[ece372349-bib-0022] Caswell, H. 2001. Matrix Population Models—Construction, Analysis, and Interpretation. 2nd ed. Sinauer Assoc., Inc.

[ece372349-bib-0023] Caswell, H. , M. Fujiwara , and S. Brault . 1999. “Declining Survival Probability Threatens the North Atlantic Right Whale.” Proceedings of the National Academy of Sciences 96, no. 6: 3308–3313. 10.1073/pnas.96.6.3308 PMC15938 10077680

[ece372349-bib-0024] Cheney, B. J. , P. M. Thompson , and L. S. Cordes . 2019. “Increasing Trends in Fecundity and Calf Survival of Bottlenose Dolphins in a Marine Protected Area.” Scientific Reports 9, no. 1: 1767. 10.1038/s41598-018-38278-9. 30741983 PMC6370779

[ece372349-bib-0026] Cordes, L. S. , and P. M. Thompson . 2014. “Mark‐Recapture Modeling Accounting for State Uncertainty Provides Concurrent Estimates of Survival and Fecundity in a Protected Harbor Seal Population.” Marine Mammal Science 30, no. 2: 691–705. 10.1111/mms.12070.

[ece372349-bib-0027] Cordes, L. S. , and P. M. Thompson . 2015. “Mark‐Resight Estimates of Seasonal Variation in Harbor Seal Abundance and Site Fidelity.” Population Ecology 57, no. 3: 467–472.

[ece372349-bib-0028] Cormack, R. M. 1964. “Estimates of Survival From the Sighting of Marked Animals.” Biometrika 51: 429–438.

[ece372349-bib-0029] Cunningham, L. 2009. “Using Computer‐Assisted Photo‐Identification and Capture‐Recapture Techniques to Monitor the Conservation Status of Harbour Seals (*Phoca vitulina*).” Aquatic Mammals 35, no. 3: 319–329.

[ece372349-bib-0030] Cunningham, L. , J. M. Baxter , I. L. Boyd , et al. 2009. “Harbour Seal Movements and Haul‐Out Patterns: Implications for Monitoring and Management.” Aquatic Conservation: Marine and Freshwater Ecosystems 19, no. 4: 398–407.

[ece372349-bib-0031] Currey, R. J. C. , S. M. Dawson , K. Schneider , et al. 2011. “Inferring Causal Factors for a Declining Population of Bottlenose Dolphins via Temporal Symmetry Capture–Recapture Modeling.” Marine Mammal Science 27, no. 3: 554–566. 10.1111/j.1748-7692.2010.00417.x.

[ece372349-bib-0033] Eberhardt, L. 1985 1985b. “Assessing the Dynamics of Wild Populations.” Journal of Wildlife Management 49: 997–1012.

[ece372349-bib-0035] Fay, R. , C. Barbraud , K. Delord , and H. Weimerskirch . 2017. “Contrasting Effects of Climate and Population Density Over Time and Life Stages in a Long‐Lived Seabird.” Functional Ecology 31, no. 6: 1275–1284. 28781406 10.1111/1365-2435.12831 PMC5518763

[ece372349-bib-0036] Friday, N. A. , T. D. Smith , P. T. Stevick , J. Allen , and T. Fernald . 2008. “Balancing Bias and Precision in Capture‐Recapture Estimates of Abundance.” Marine Mammal Science 24: 253–275. 10.1111/j.1748-7692.2008.00187.x.

[ece372349-bib-0037] Fritz, L. W. , R. Towell , T. S. Gelatt , D. S. Johnson , and T. R. Loughlin . 2014. “Recent Increases in Survival of Western Steller Sea Lions in Alaska and Implications for Recovery.” Endangered Species Research 26: 13–24. 10.3354/esr00634.

[ece372349-bib-0118] Gaillard, J.‐M. , M. Festa‐Bianchet , and N. G. Yoccoz . 1998. “Population Dynamics of Large Herbivores: Variable Recruitment With Constant Adult Survival.” Trends in Ecology & Evolution 13, no. 2: 58–63. 10.1016/s0169-5347(97)01237-8. 21238201

[ece372349-bib-0038] Galatius, A. , S. Brasseur , T. Hamm , et al. 2023. “Survey Results of Harbour Seals in the Wadden Sea in 2023.”

[ece372349-bib-0039] Gimenez, O. , J.‐D. Lebreton , R. Choquet , and R. Pradel . 2018. “R2ucare: An R Package to Perform Goodness‐of‐Fit Tests for Capture‐Recapture Models.” *bioRxiv*. 10.1101/192468.

[ece372349-bib-0041] Greig, D. J. 2011. “Health, Disease, Mortality and Survival in Wild and Rehabilitated Harbor Seals (*Phoca vitulina*) in San Francisco Bay and Anlong the Central California Coast.” PhD thesis, University of St Andrews, St Andrews, UK 197 pp.

[ece372349-bib-0042] Hall, A. , and E. Frame . 2010. “Evidence of Domoic Acid Exposure in Harbour Seals From Scotland: A Potential Factor in the Decline in Abundance?” Harmful Algae 9: 489–493. 10.1016/j.hal.2010.03.004.

[ece372349-bib-0043] Hall, A. , R. Hewitt , and M. Arso Civil . 2020. “Determining Pregnancy Status in Harbour Seals Using Progesterone Concentrations in Blood and Blubber.” General and Comparative Endocrinology 295: 113529. 10.1016/j.ygcen.2020.113529. 32522487

[ece372349-bib-0044] Hall, A. , and Workshop participants . 2012. “Workshop Report on Decline in Abundance of Harbour Seals Around the Coast of Scotland and Discussion of Mitigation and Management Measures.” Sea Mammal Research Unit, University of St Andrews, Report to Scottish Government, no. CSD 1 & CSD 2, St Andrews, 11 pp. Microsoft Word–CDS1 & 2 Report for Web.docx.

[ece372349-bib-0045] Hall, A. J. , J. L. Kershaw , S. Fraser , et al. 2024. “Estimating the Risks of Exposure to Harmful Algal Toxins Among Scottish Harbour Seals.” Harmful Algae 136: 102653. 38876527 10.1016/j.hal.2024.102653

[ece372349-bib-0046] Hall, A. J. , B. Mackey , J. L. Kershaw , and P. Thompson . 2019. “Age–Length Relationships in UK Harbour Seals During a Period of Population Decline.” Aquatic Conservation: Marine and Freshwater Ecosystems 29: 61–70.

[ece372349-bib-0047] Hall, A. J. , and G. O. Thomas . 2007. “Polychlorinated Biphenyls, DDT, Polybrominated Diphenyl Ethers and Organic Pesticides in United Kingdom Harbor Seals—Mixed Exposures and Thyroid Homeostasis.” Environmetnal Toxicology & Chemistry 26: 851–861. 10.1897/06-310R.1. 17521129

[ece372349-bib-0049] Hanson, N. , D. Thompson , C. Duck , S. Moss , and M. Lonergan . 2013. “Pup Mortality in a Rapidly Declining Harbour Seal ( *Phoca vitulina* ) Population.” PLoS One 8, no. 11: e80727. 24312239 10.1371/journal.pone.0080727 PMC3842331

[ece372349-bib-0050] Harding, K. C. , M. Fujiwara , Y. Axberg , and T. Härkönen . 2005. “Mass‐Dependent Energetics and Survival in Harbour Seal Pups.” Functional Ecology 19: 129–135.

[ece372349-bib-0051] Härkönen, T. , and K. C. Harding . 2001. “Spatial Structure of Harbour Seal Populations and the Implications Thereof.” Canadian Journal of Zoology 79, no. 12: 2115–2127. 10.1139/z01-172.

[ece372349-bib-0052] Härkönen, T. , K. C. Hårding , and S. G. Lunneryd . 1999. “Age‐and Sex‐Specific Behaviour in Harbour Seals *Phoca vitulina* Leads to Biased Estimates of Vital Population Parameters.” Journal of Applied Ecology 36, no. 5: 825–841.

[ece372349-bib-0053] Härkönen, T. , and M. P. Heide‐Jørgensen . 1990. “Comparative Life Histories of East Atlantic and Other Harbour Seal Populations.” Ophelia 32, no. 3: 211–235. 10.1080/00785236.1990.10422032.

[ece372349-bib-0054] Hastings, K. K. , T. S. Gelatt , J. M. Maniscalco , et al. 2023. “Reduced Survival of Steller Sea Lions in the Gulf of Alaska Following Marine Heatwave.” Frontiers in Marine Science 10: 1127013.

[ece372349-bib-0055] Hastings, K. K. , R. J. Small , and G. W. Pendleton . 2012. “Sex‐and Age‐Specific Survival of Harbor Seals ( *Phoca vitulina* ) From Tugidak Island, Alaska.” Journal of Mammalogy 93: 1368–1379. 10.1644/11-MAMM-A-291.1.

[ece372349-bib-0056] Herrera Fuchs, Y. , G. J. Edgar , N. S. Barrett , et al. 2025. “Contrasting Population Trajectories of Temperate Reef Fishes and Invertebrates Following Seasonal and Multi‐Decadal Temperature Change.” Global Change Biology 31, no. 5: e70233. 10.1111/gcb.70233. 40356052 PMC12069756

[ece372349-bib-0057] Huggins, R. M. 1989. “On the Statistical Analysis of Capture Experiments.” Biometrika 76, no. 1: 133–140.

[ece372349-bib-0058] Jacobson, E. K. , M. Arso Civil , B. J. Cheney , et al. n.d. (in prep). “Integrated Population Models to Investigate Harbour Seal Population Trajectories in Scotland.”

[ece372349-bib-0059] Jemison, L. A. , and B. P. Kelly . 2001. “Pupping Phenology and Demography of Harbor Seals ( *Phoca vitulina richardsi* ) on Tugidak Island, Alaska.” Marine Mammal Science 17: 585–600.

[ece372349-bib-0061] Jensen, S.‐K. , J.‐P. Lacaze , G. Hermann , et al. 2015. “Detection and Effects of Harmful Algal Toxins in Scottish Harbour Seals and Potential Links to Population Decline.” Toxicon 97: 1–14. 10.1016/j.toxicon.2015.02.002. 25666120

[ece372349-bib-0062] Jolly, G. M. 1965. “Explicit Estimates From Capture‐Recapture Data With Both Death and Immigration‐Stochastic Model.” Biometrika 52: 225–247. 14341276

[ece372349-bib-0063] Kendall, W. L. , J. E. Hines , and J. D. Nichols . 2003. “Adjusting Multistate Capture‐Recapture Models for Misclassification Bias: Manatee Breeding Proportions.” Ecology 84: 1058–1066. 10.1890/0012-9658(2003)084[1058:AMCMFM]2.0.CO;2.

[ece372349-bib-0064] Kershaw, J. L. , C. A. Ramp , R. Sears , et al. 2021. “Declining Reproductive Success in the Gulf of St. Lawrence's Humpback Whales (*Megaptera novaeangliae*) Reflects Ecosystem Shifts on Their Feeding Grounds.” Global Change Biology 27, no. 5: 1027–1041. 10.1111/gcb.15466 33368899

[ece372349-bib-0065] Laake, J. L. 2013. “RMark: An R Interface for Analysis of Capture‐Recapture Data With MARK. AFSC Processed Rep 2013‐01, 25p.” Alaska Fisheries Science Cent., NOAA, Natl. Mar. Fish. Serv., 7600 Sand Point Way NE, Seattle WA 98115.

[ece372349-bib-0066] Lacy, R. C. 2000. “Structure of the VORTEX Simulation Model for Population Viability Analysis.” Ecological Bulletins 48: 191–203.

[ece372349-bib-0067] Langley, I. 2024. “Interspecific Interactions: Investigating the Role of Grey Seals in the Harbour Seal Decline.” PhD thesis (Unpublished), University of St Andrews. 10.17630/sta/1228.

[ece372349-bib-0119] Langley, I. , A. Brownlow , and D. J. F. Russell . 2025. “Intraguild Predation in Sympatric Seals and the Effect on a Declining Population.” Journal of Animal Ecology. 10.1111/1365-2656.70152. PMC12673244 41077884

[ece372349-bib-0069] Langley, I. , E. Hague , and M. Arso Civil . 2021. “Assessing the Performance of Open‐Source, Semi‐Automated Pattern Recognition Software for Harbour Seal (*P*. *v*. *Vitulina*) Photo ID.” Mammalian Biology 10: 973–982. 10.1007/s42991-021-00165-8.

[ece372349-bib-0070] Lebreton, J.‐D. , K. P. Burnham , J. Clobert , and D. R. Anderson . 1992. “Modeling Survival and Testing Biological Hypotheses Using Marked Animals: A Unified Approach With Case Studies.” Ecological Monographs 62, no. 1: 67–118. 10.2307/2937171.

[ece372349-bib-0073] Lonergan, M. , C. Duck , D. Thompson , B. Mackey , L. Cunningham , and I. Boyd . 2007. “Using Sparse Survey Data to Investigate the Declining Abundance of British Harbour Seals.” Journal of Zoology 271, no. 3: 261–269.

[ece372349-bib-0074] Lonergan, M. , C. D. Duck , S. Moss , C. Morris , and D. Thompson . 2013. “Rescaling of Aerial Survey Data With Information From Small Numbers of Telemetry Tags to Estimate the Size of a Declining Harbour Seal Population.” Aquatic Conservation: Marine and Freshwater Ecosystems 23: 135–144.

[ece372349-bib-0115] Lonergan, M. , A. Hall , H. Thompson , P. M. Thompson , P. Pomeroy , and J. Harwood . 2010. “Comparison of the 1988 and 2002 Phocine Distemper Epizootics in British Harbour Seal *Phoca vitulina* Populations.” Diseases of Aquatic Organisms 88: 183–188. 10.3354/dao02153. 20377007

[ece372349-bib-0075] Lowry, L. F. , K. J. Frost , J. M. Ver Hoep , and R. A. Delong . 2001. “Movements of Satellite‐Tagged Subadult and Adult Harbor Seals in Prince William Sound, Alaska.” Marine Mammal Science 17, no. 4: 835–861.

[ece372349-bib-0076] Lydersen, C. , and K. M. Kovacs . 2005. “Growth and Population Parameters of the World's Northernmost Harbour Seals *Phoca vitulina* Residing in Svalbard, Norway.” Polar Biology 28, no. 2: 156–163. 10.1007/s00300-004-0656-7.

[ece372349-bib-0077] Mackey, B. L. , J. W. Durban , S. J. Middlemas , and P. M. Thompson . 2008. “A Bayesian Estimate of Harbour Seal Survival Using Sparse Photo‐Identification Data.” Journal of Zoology 274, no. 1: 18–27. 10.1111/j.1469-7998.2007.00352.x.

[ece372349-bib-0078] Manugian, S. C. , D. Greig , D. Lee , et al. 2017. “Survival Probabilities and Movements of Harbor Seals in Central California.” Marine Mammal Science 33, no. 1: 154–171. 10.1111/mms.12350.

[ece372349-bib-0079] Matthiopoulos, J. , L. Cordes , B. Mackey , et al. 2014. “State‐Space Modelling Reveals Proximate Causes of Harbour Seal Population Declines.” Oecologia 174: 151–162. 10.1007/s00442-013-2764-y. 24036987

[ece372349-bib-0080] McConnell, B. , S. Smout , and M. Wu . 2017. “Modelling Harbour Seal Movements.” Scottish Marine and Freshwater Science 8: 1–33. 10.7489/1998-1.

[ece372349-bib-0081] McMahon, C. R. , M. N. Bester , H. R. Burton , M. A. Hindell , and C. J. A. Bradshaw . 2005. “Population Status, Trends and a Re‐Examination of the Hypotheses Explaining the Recent Declines of the Southern Elephant Seal *Mirounga leonina* .” Mammal Review 35, no. 1: 82–100. 10.1111/j.1365-2907.2005.00055.x.

[ece372349-bib-0082] Nichols, J. D. , W. L. Kendall , J. E. Hines , and J. A. Spendelow . 2004. “Estimation of Sex‐Specific Survival From Capture‐Recapture Data When Sex Is not Always Known.” Ecology 85: 3192–3201. 10.1890/03-0578.

[ece372349-bib-0083] Olsen, M. T. , V. Islas , J. A. Graves , et al. 2017. “Genetic Population Structure of Harbour Seals in the United Kingdom and Neighbouring Waters.” Aquatic Conservation: Marine and Freshwater Ecosystems 27, no. 4: 839–845.

[ece372349-bib-0084] Pendleton, G. W. , K. W. Pitcher , L. W. Fritz , et al. 2006. “Survival of Steller Sea Lions in Alaska: A Comparison of Increasing and Decreasing Populations.” Canadian Journal of Zoology 84, no. 8: 1163–1172.

[ece372349-bib-0085] Pitcher, K. W. , and D. G. Calkins . 1979. “Biology of the Harbour Seal, Phoca Vitulina Richardsi, in the Gulf of Alaska Final Report, Outer Continental Shelf Environmental Assessment Program Research Unit 229, Contract Number 03‐5‐002‐69, Issue.”

[ece372349-bib-0086] Pradel, R. 1993. Flexibility in Survival Analysis From Recapture Data: Handling Trap‐Dependence, 29–37. Birkhauser Verlag.

[ece372349-bib-0087] Pradel, R. , J. E. Hines , J. D. Lebreton , and J. D. Nichols . 1997. “Capture‐Recapture Survival Models Taking Account of Transients.” Biometrics 53, no. 1: 60–72.

[ece372349-bib-0088] Pradel, R. , and A. Sanz‐Aguilar . 2012. “Modeling Trap‐Awareness and Related Phenomena in Capture‐Recapture Studies.” PLoS One 7: e32666. 22396787 10.1371/journal.pone.0032666 PMC3292565

[ece372349-bib-0089] R Core Team . 2023. R: A Language and Environment for Statistical Computing. R Foundation for Statistical Computing. R Foundation for Statistical Computing. https://www.R‐project.org/.

[ece372349-bib-0090] Reijnders, P. J. , E. H. Ries , S. Tougaard , et al. 1997. “Population Development of Harbour Seals *Phoca vitulina* in the Wadden Sea After the 1988 Virus Epizootic.” Journal of Sea Research 38, no. 1–2: 161–168.

[ece372349-bib-0091] Rubertus, R. P. 1983. “Observations on a Colony of Common Seals (*Phoca vitulina*) on Burray (Orkney) in July 1982.” Final Report to the European Communities (Projectnumber ENV616N), RIN, Arnhem, The Netherlands.

[ece372349-bib-0092] Russell, D. J. F. , C. D. Duck , C. D. Morris , N. G. Riddoch , and D. Thompson . 2024. “Trends in Seal Abundance and Grey Seal Pup Production.” BP 24/03 in Scientific Advice on Matters Related to the Management of Seal Populations: 2024.

[ece372349-bib-0093] Russell, D. J. F. , B. T. McClintock , J. Matthiopoulos , et al. 2015. “Intrinsic and Extrinsic Drivers of Activity Budgets in Sympatric Grey and Harbour Seals.” Oikos 124: 1462–1472. 10.1111/oik.01810.

[ece372349-bib-0095] Schleimer, A. , C. Ramp , J. Delarue , et al. 2019. “Decline in Abundance and Apparent Survival Rates of Fin Whales (*Balaenoptera physalus*) in the Northern Gulf of St. Lawrence.” Ecology and Evolution 9, no. 7: 4231–4244. 10.1002/ece3.5055. 31016001 PMC6468087

[ece372349-bib-0096] SCOS . 2020. “Scientific Advice on Matters Related to the Management of Seal Populations: 2020.”

[ece372349-bib-0097] SCOS . 2024. “Scientific Advice on Matters Related to the Management of Seal Populations: 2024.”

[ece372349-bib-0098] Seber, G. A. F. 1965. “A Note on Multiple‐Recapture Census.” Biometrika 52: 249–259. 14341277

[ece372349-bib-0101] Sutherland, J. C. 2024. “Killer Whale Predation of Seals in the Inshore Waters of Shetland: Investigating the Ecological Drivers and Consequences of an Apex Predator–Prey Interaction.” PhD thesis (unpublished), University of St Andrews. 10.17630/sta/1116.

[ece372349-bib-0102] Svensson, C. J. 2012. “Seal Dynamics on the Swedish West Coast: Scenarios of Competition as Baltic Grey Seal Intrude on Harbour Seal Territory.” Journal of Sea Research 71: 9–13. 10.1016/j.seares.2012.03.005.

[ece372349-bib-0103] Thompson, D. , C. Duck , C. Morris , and D. J. F. Russell . 2019. “The Status of Harbour Seals (*Phoca vitulina*) in the UK.” Aquatic Conservation: Marine and Freshwater Ecosystems 29, no. S1: 40–60. 10.1002/aqc.3110.

[ece372349-bib-0104] Thompson, P. M. , K. M. Kovacs , and B. J. McCocConnell . 1994. “Natal Dispersal of Harbour Seals (*Phoca vitulina*) From Breeding Sites in Orkney, Scotland.” Journal of Zoology 234, no. 4: 668–673.

[ece372349-bib-0105] Thompson, P. M. , D. Miller , R. Cooper , and P. S. Hammond . 1994. “Changes in the Distribution and Activity of Female Harbour Seals During the Breeding Season: Implications for Their Lactation Strategy and Mating Patterns.” Journal of Animal Ecology 63, no. 1: 24–30. 10.2307/5579.

[ece372349-bib-0116] Thompson, P. , and P. Rothery . 1987. “Age and Sex Differences in the Timing of Moult in the Common Seal, *Phoca vitulina* .” Journal of Zoology 212, no. 4: 597–603. 10.1111/j.1469-7998.1987.tb05958.x.

[ece372349-bib-0106] Thompson, P. M. , D. J. Tollit , D. Wood , H. M. Corpe , P. S. Hammond , and A. Mackay . 1997. “Estimating Harbour Seal Abundance and Status in an Estuarine Habitat in North‐East Scotland.” Journal of Applied Ecology 34: 43–52.

[ece372349-bib-0107] Thompson, P. M. , S. Van Parijs , and K. M. Kovacs . 2001. “Local Declines in the Abundance of Harbour Seals: Implications for the Designation and Monitoring of Protected Areas.” Journal of Applied Ecology 38, no. 1: 117–125. 10.1046/j.1365-2664.2001.00571.x.

[ece372349-bib-0108] Trites, A. W. 2021. “Behavioral Insights Into the Decline and Natural History of Steller Sea Lions.” In Ethology and Behavioral Ecology of Otariids and the Odobenid, edited by C. Campagna and R. Harcourt , 489–519. Springer International Publishing. 10.1007/978-3-030-59184-7_23.

[ece372349-bib-0109] van Neer, A. , L. F. Jensen , and U. Siebert . 2015. “Grey Seal ( *Halichoerus grypus* ) Predation on Harbour Seals ( *Phoca vitulina* ) on the Island of Helgoland, Germany.” Journal of Sea Research 97: 1–4.

[ece372349-bib-0110] Warlick, A. J. , G. K. Himes Boor , T. L. McGuire , et al. 2024. “Identifying Demographic and Environmental Drivers of Population Dynamics and Viability in an Endangered Top Predator Using an Integrated Model.” Animal Conservation 27, no. 2: 240–252. 10.1111/acv.12905.

[ece372349-bib-0111] White, G. C. 2014. “Robust Design Multi‐State With State Uncertainty.” https://sites.warnercnr.colostate.edu/gwhite/robust‐design‐multistate‐state‐uncertainty/.

[ece372349-bib-0112] White, G. C. , and K. P. Burnham . 1999. “Program MARK: Survival Estimation From Populations of Marked Animals.” Bird Study 46: 120–139.

[ece372349-bib-0113] Williams, B. K. , J. D. Nichols , and M. J. Conroy . 2002. Analysis and Management of Animal Populations: Modeling, Estimation, and Decision Making. Academic Press.

[ece372349-bib-0114] Wilson, L. J. , and P. S. Hammond . 2019. “The Diet of Harbour and Grey Seals Around Britain: Examining the Role of Prey as a Potential Cause of Harbour Seal Declines.” Aquatic Conservation: Marine and Freshwater Ecosystems 29: 71–85.

